# Opioidergic Signaling—A Neglected, Yet Potentially Important Player in Atopic Dermatitis

**DOI:** 10.3390/ijms23084140

**Published:** 2022-04-08

**Authors:** Dorottya Ádám, József Arany, Kinga Fanni Tóth, Balázs István Tóth, Attila Gábor Szöllősi, Attila Oláh

**Affiliations:** 1 Department of Physiology, Faculty of Medicine, University of Debrecen, 4032 Debrecen, Hungary; adam.dorottya@med.unideb.hu (D.Á.); arany.jozsef@med.unideb.hu (J.A.); kingafanni.tth@gmail.com (K.F.T.); toth.istvan@med.unideb.hu (B.I.T.); 2 Doctoral School of Molecular Medicine, University of Debrecen, 4032 Debrecen, Hungary; 3 Department of Immunology, Faculty of Medicine, University of Debrecen, 4032 Debrecen, Hungary; szollosi.attila@med.unideb.hu

**Keywords:** atopic dermatitis (AD), cutaneous barrier, δ-opioid receptor (DOR), inflammation, itch, κ-opioid receptor (KOR), keratinocyte, mast cell, µ-opioid receptor (MOR), nociceptin/orphanin FQ (NOP) receptor, opioid, skin

## Abstract

Atopic dermatitis (AD) is one of the most common skin diseases, the prevalence of which is especially high among children. Although our understanding about its pathogenesis has substantially grown in recent years, and hence, several novel therapeutic targets have been successfully exploited in the management of the disease, we still lack curative treatments for it. Thus, there is an unmet societal demand to identify further details of its pathogenesis to thereby pave the way for novel therapeutic approaches with favorable side effect profiles. It is commonly accepted that dysfunction of the complex cutaneous barrier plays a central role in the development of AD; therefore, the signaling pathways involved in the regulation of this quite complex process are likely to be involved in the pathogenesis of the disease and can provide novel, promising, yet unexplored therapeutic targets. Thus, in the current review, we aim to summarize the available potentially AD-relevant data regarding one such signaling pathway, namely cutaneous opioidergic signaling.

## 1. Introduction

Appropriate cutaneous barrier functions (i.e., physicochemical, immunological, and microbiological barriers) are indispensable for survival, and their disturbances can lead to extremely prevalent diseases, such as atopic dermatitis (AD). Indeed, AD is estimated to affect ~15–30% of children, 2–10% of the adults, and 1–3% of the elderly population [1,2,3,4]. Hence, it is one of the most common diseases of the human integumentary system. It substantially impairs the quality of life of millions worldwide and represents a significant burden to both the affected individuals and to society [1,2,3,4]. Although several details of its pathogenesis are still unknown, the research efforts of the last decades have paved the way for a number of novel therapeutic approaches that are currently in various phases of drug development. These agents include, but are not limited to, phosphodiesterase-4 inhibitors, H_4_ histamine receptor antagonists, interleukin (IL)-4/IL-13 (dupilumab), IL-17A (secukinumab) and IL-22 (fezakinumab) inhibitors, IL-31 receptor blockers (nemolizumab), boron molecules, Janus kinase inhibitors, modulators of cannabinoid signaling, etc. [5,6,7,8,9,10,11,12,13]. Moreover, modulation of the cutaneous adipokine signaling, as well as the administration of native and/or appropriately engineered extracellular vesicles (EVs), were also suggested to be explored in the treatment of AD [14,15].

### 1.1. Key Aspects of the Pathogenesis of AD

AD is a complex disease with multiple different clinical subtypes, and its pathogenesis is still incompletely understood. AD can be classified into two major endotypes based on both symptoms and pathogenesis: the more common “extrinsic” (high serum IgE levels, eosinophilia, atopic background, greater filaggrin (FLG) mutation rate) and the rarer “intrinsic” (normal serum IgE level, delayed onset, female predominance, more preserved barrier function, increased prevalence of metal contact hypersensitivity, lack of other atopic background) types. Moreover, to make things more complex, patients from the same endotypes were found to exhibit certain dissimilarities across different races in the immune polarization and epidermal barrier function [16], and a wide variety of environmental factors were also shown to contribute to the pathogenesis of the disease [17,18].

Considering this versatility, it is not surprising that multiple hypotheses (e.g., “outside to inside” and “inside to outside”) have been formulated to describe and explain the chain of events leading to the development of symptoms in AD. In light of the recent data, a combined approach, namely the “outside to inside and back to outside” hypothesis, seems to be the most appropriate, suggesting that in most cases, the primary disorder affects the epidermal barrier structure and function. This results in a pathological immune response that provokes further secondary barrier abnormalities [19,20,21].

It is well known that disturbance of every element of the complex cutaneous barrier (i.e., physicochemical, microbiological, and immunological barriers) [22,23] contributes to the development of the symptoms in AD; however, barrier dysfunction is not the sole player in the pathogenesis. Indeed, among others, disturbed differentiation of epidermal keratinocytes and hence defective physicochemical barrier due to e.g., genetic predisposition (e.g., FLG mutations) or other factors [1,2,24], abnormal skin lipid profile, as well as pathological sebaceous gland density and function [25,26,27], elevated skin surface pH [28], pathological alterations in the cutaneous microbiota [29,30,31,32], certain dietary factors [33,34], altered gut microbiota (“gut–skin” or “gut–brain–skin” axis) [34,35,36,37], abnormal vascular responses (white dermographism) [27,38,39,40], mediator secretion of sensory nerve endings leading to neurogenic inflammation [41,42,43], as well as the pathological T_h_2/T_h_22-dominated immune responses [1,2], defects of the innate immune system (e.g., dysfunction of certain pathogen-associated molecular-pattern-recognizing receptors and anti-microbial peptides, defects in the natural killer (NK) cell functions, decreased responsiveness of neutrophils to chemoattractants, altered mast cell (MC) activity, etc.) [44,45], as well as the detrimental consequences of the “itch–scratch cycle” [46,47], all contribute to the development of AD, and it is obvious that covering all these aspects in details would extend far beyond a single paper.

As mentioned above, although the root cause of the disease is not entirely clear, dysfunction of epidermal keratinocytes definitely plays a central role in the process. Indeed, proper differentiation of these cells is essential to form the physicochemical barrier [22,23,48]. Moreover, keratinocytes can serve as “universal sensors” and potent regulators that are capable of not only detecting a wide variety of environmental signals (including, but not limited to, temperature, osmolarity, mechanical stimuli, UV light, presence of various microbes, etc.) [49,50,51,52,53] but also of “processing” and “translating” them to sensory neurons or professional immune cells by releasing numerous intercellular messengers (EVs, pro- and anti-inflammatory cytokines and chemokines, itch mediators, etc.), as well as anti-microbial peptides [46,48,49,50,51,52,53,54,55,56,57,58,59,60,61,62,63]. Thus, a better understanding of the biology of epidermal keratinocytes, as well as of the cross-talk between them and various members of the cutaneous immune system, holds out the promise to highlight hidden aspects of barrier-disturbance and/or cutaneous-inflammation-accompanied skin diseases and thereby to unveil novel, efficient therapeutic opportunities. Following the above logic, it seems to be safe to assume that appropriate intercellular communication is crucially important in the maintenance of skin homeostasis and in the prevention of the onset of AD. Thus, in this paper, we intend to review the currently available evidence related to the role of a potentially “AD-relevant” intercellular signaling system, namely opioidergic signaling, and to highlight how these pieces of information could translate into novel therapeutic approaches in the future.

### 1.2. Overview of Opioidergic Signaling

**Figure 1:**
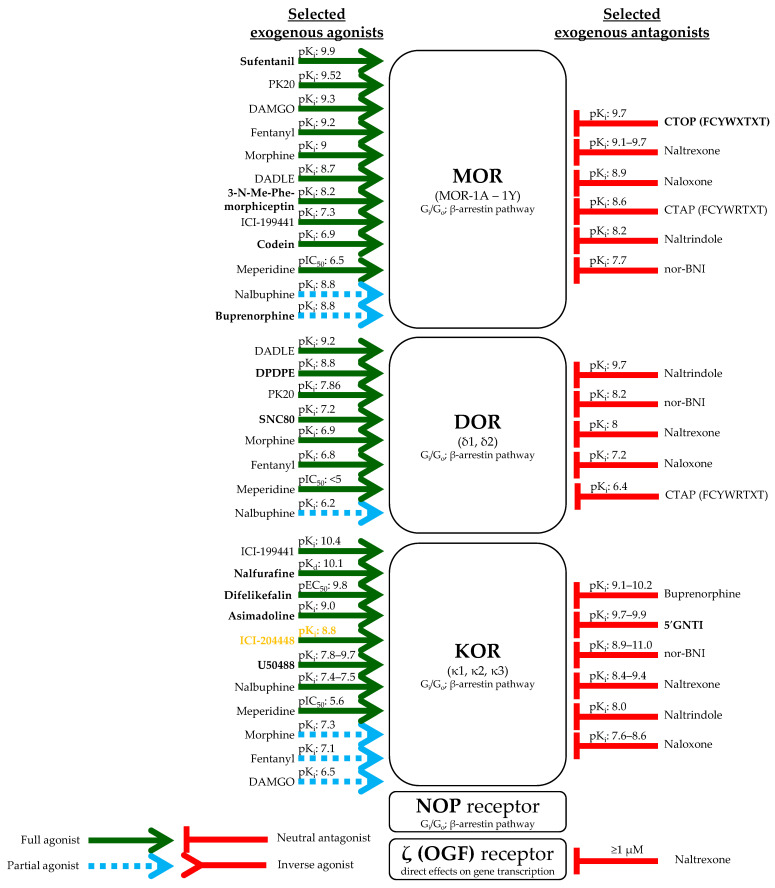
Overview of the major opioid receptors and their most important exogenous ligands mentioned in the text. Affinity data are obtained from the IUPHAR Guide to Pharmacology database (accessed on 5 December 2021) or (in case of PK20) from Ref [82]. When available, affinities for the human receptors are shown. If such data were not available, animal data are given (indicated by **yellow fonts**). **Bold characters** highlight apparently selective compounds (i.e., compounds that have no other targets according to the said database). Note that, unlike the other opioid receptors, ζ (OGF) receptor is normally expressed in the nuclear envelope. Upon binding OGF, it translocates to the nucleus, where it directly regulates gene expression [79]. Please note that ***we only provide affinity but not efficacy data*** of the substances.

The opioidergic system is a complex intercellular signaling system composed of endogenous receptors and ligands [64,65]. It is one of the most studied endogenous analgesic systems, but it has been shown to be involved in the regulation of a wide variety of biological processes (e.g., immune response, regulation of gastrointestinal motility, respiration, etc.) as well [66,67,68]. The most important opioid receptors are the μ- (MOR), κ- (KOR), δ- (DOR), and nociceptin/orphanin FQ (NOP) receptors, which all belong to the G-protein-coupled 7-TM receptor superfamily. They are usually (but not exclusively) coupled with G_i_/G_o_ proteins; hence, their activation is typically followed by a reduction in the cAMP level. Moreover, they can also activate the β-arrestin pathways [65], and they exhibit clinically relevant ligand-dependent signaling bias [69,70,71] (Figure 1). Last, but not least, it should also be noted that β and γ subunits of the aforementioned G proteins may modulate the activity of several ion channels. For example, transient receptor potential melastatin (TRPM)-3 ion channels are known to be key players in the detection of noxious heat, as well as in inflammatory thermal hyperalgesia, while they are not involved in mediating itch evoked by endogenous pruritogens [72,73]. Importantly, it has recently been shown that activity of TRPM3 in somatosensory neurons is tightly regulated by MOR through the signaling of G_βγ_ proteins that directly bind to TRPM3 and thereby reduce TRPM3-mediated pain sensation [74,75,76,77].

Here, it has to be noted that, just like other G-protein-coupled receptors, opioid receptors are also able to form functional homo- and heterodimers. Although details of the biological properties of these multimers are to be explored in future studies, it seems to be safe to assume that the functional characteristics of such complexes are somewhat different from the monomeric receptor forms, which can contribute to the signaling versatility of this system [65]. Finally, it is noteworthy that, due to e.g., alternative splicing, MOR, KOR, and DOR may have different subtypes, namely MOR-1A to MOR-1Y (MOR), the putative κ1, κ2, and κ3 (KOR), and the putative δ1 and δ2 (DOR) (Figure 1) [65,78]. Although it is still unclear whether and how the functions of these putative receptor subtypes differ from each other, they (or at least some of them) may have functional relevance, which is to be explored in future studies [65].

Besides the four major opioid targets mentioned above, several other receptors have also been suggested to belong to this family. These putative receptors include “lambda” (λ) and “sigma” (σ) receptors, as well as the β-endorphin-sensitive “epsilon” (ε) and the Met-enkephalin (also known as “opioid growth factor” (OGF))-sensitive “zeta” (ζ or OGF) receptors [65,79,80]. Because λ and ε receptors have not been cloned or sequenced yet, and σ receptors are no longer considered to be opioid receptors [65], in the current paper, we only mention potentially AD-relevant data of ζ (OGF) receptors (Figure 1).

**Figure 2:**
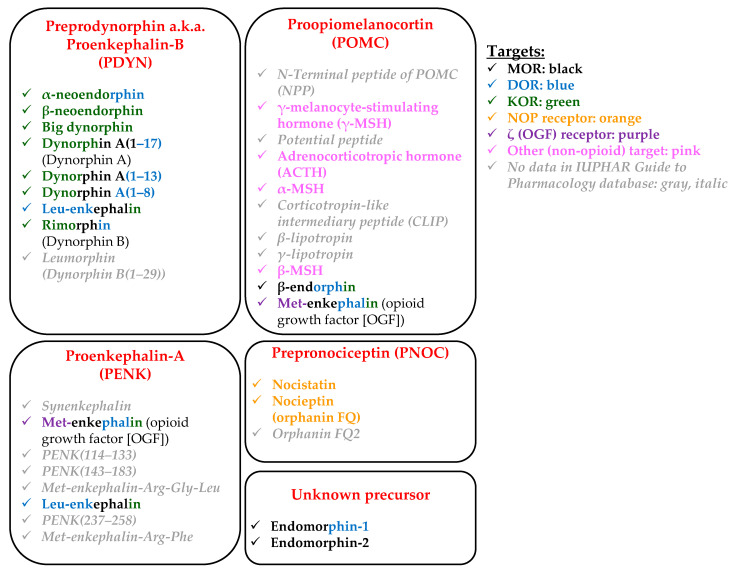
Overview of the origin and receptor affinity of the most important endogenous opioids. The color code indicates the relevant targets of each molecule. When one molecule can activate more than one receptor, the order of the colors from the left to the right represents the order of the affinity toward each receptor. For example, the order of colors in case of “**β-endorphin**” (**black-blue-green**) indicates that its affinity is the highest to human MOR, it is less to DOR, and even less to KOR. Affinity data are obtained from the IUPHAR Guide to Pharmacology database (accessed on 5 December 2021) and from Ref [83]. Importantly, lack of a specific color does not necessarily mean lack of the respective binding, but rather *lack of experimental evidence* of the said binding. Please note that ***we only provide affinity but not efficacy data*** of the substances.

In the past decades, several endogenous and exogenous ligands of the above receptors have been identified and characterized. Although they can usually concentration dependently cross-activate multiple opioid receptors (Figure 2), the ligands can be classified according to their “primary” receptor preference. Indeed, β-endorphin and endomorphins are mostly MOR-selective ligands, Leu-enkephalin favors DOR, dynorphins are rather KOR-selective molecules, whereas nociceptin and Met-enkephalin (OGF) preferentially activate NOP and ζ (OGF) receptors, respectively [65,81].

Endogenous opioid peptides are derived from four major precursors, namely proopiomelanocortin (POMC; precursor of β-endorphin), proenkephalin-A (PENK; precursor of enkephalins), preprodynorphin (PDYN; also known as proenkephalin-B; precursor of dynorphins and neoendorphins), and pronociceptin/orphanin FQ (precursor of nociceptin/orphanin FQ and nocistatin) [81] (Figure 2). Although their synthesis/activation and degradation are not completely explored, several enzymes are known to be involved in the synthesis and degradation of the above endogenous opioids. For example, prohormone convertase (PC)-1/3 and PC-2 are sequentially involved in the production [84], whereas carboxypeptidase E (CPE) is required for the activation of β-endorphin [85,86].

It should also be noted that POMC-derived peptides include not only opioids but several other biologically active substances as well, including adrenocorticotropic hormone (ACTH), α-, β-, and γ-melanocyte-stimulating hormone (α-, β-, and γ-MSH) or γ-lipotropin, etc., highlighting that endogenous opioid signaling may interact with several other signaling pathways [81]. Last, but not least, it is worth mentioning that biological effects of the endogenous opioids can be modulated by other peptides (e.g., melanocyte inhibiting factor (MIF)-related peptides, cholecystokinin (CCK), neuropeptide FF (NPFF), etc.), collectively referred to as “anti-opioid peptides” [87,88].

## 2. Role of the Opioidergic Signaling in AD

Skin is not only a passive barrier that protects our body, but it is rather an active neuroendocrinoimmune organ [89,90,91], and a growing body of evidence suggests that opioidergic signaling plays an important role in regulating various aspects of its biology [92,93,94,95,96,97]. As mentioned above, in the current paper, we intend to briefly summarize the potentially AD-relevant aspects of opioidergic signaling.

### 2.1. Expression of the Opioidergic System in the Skin

#### 2.1.1. MOR, DOR, KOR, as Well as Their Major Endogenous Ligands, Are Present in the Skin

In spite of some conflicting data (see below), the vast majority of the studies agree that the most studied opioid receptors (i.e., MOR, DOR, and KOR) as well as their most important endogenous ligands (e.g., β-endorphin, several different dynorphins and enkephalins, etc.) are expressed in multiple cell types of healthy human skin [93,95,98].

The very first report claiming that MOR is expressed in the human epidermis at the mRNA (in situ hybridization) and protein levels (immunofluorescent labeling) was published in 1998 [99]. Moreover, it was also shown that 48 h treatment of organ-cultured human skin specimens (d = 4 mm; thickness ~ 0.5 mm) of healthy individuals with naltrexone (100 μM) or β-endorphin (10 μM) downregulated the expression of MOR in the epidermis [99]. Likewise, upon binding to MOR, fluorescently labeled endomorphin-1 (200 nM) was shown to be rapidly (in 10–60 min) internalized by cultured, immortalized human N/TERT-1 keratinocytes [100]. These data indicated that binding of certain ligands could induce a dynamic trafficking of MOR between the cell membrane and the intracellular compartments, ultimately leading to the downregulation of the receptor pool that is available for further ligands.

Later, it was also demonstrated that MOR was predominantly expressed in the basal and suprabasal layers, whereas its major (yet non-specific; Figure 2) ligand, i.e., β-endorphin, could be detected in every epidermal layer of the human skin (mammary region (samples were obtained from patients undergoing breast reduction)). Besides, epidermal non-myelinated nerve fibers were also shown to express MOR, and some nerve fibers were positive for β-endorphin as well [101].

MOR and KOR were also found to be expressed in cultured primary human epidermal keratinocytes, as well as in HaCaT keratinocytes (a widely used human keratinocyte cell line [102]) [103]. Moreover, β-endorphin, dynorphin A (1-8), as well as dynorphin A (1-17), were detected in the epidermis of healthy volunteers. Dynorphins were present in the whole epidermis, whereas, in contrast to its previously reported expression pattern [101], β-endorphin was only expressed in the suprabasal layers (abdominal skin of male patients) [103]. Although the authors did not attempt to solve this contradiction, one might speculate that it may reflect a regional or a sex difference in the expression pattern of β-endorphin, since the two studies utilized skin specimens from different regions (breast vs. abdominal skin).

Last, but not least, DOR- as well as PENK-derived peptides (Met- and/or Leu-enkephalin) were also found to be expressed in the epidermis, predominantly in the suprabasal and granular layers [104,105].

#### 2.1.2. NOP Receptor, as Well as ζ (OGF) Receptor, Are Expressed in the Skin

NOP receptor was found to be expressed in all epidermal layers but not in the dermis of the human skin [106]. Moreover, it was significantly downregulated (as determined by immunofluorescent labeling) in epidermal keratinocytes of pachyonychia congenita (a keratin disease caused by, among others, mutations in keratin (K)-6, characterized by palmoplantar keratoderma and neuropathic pain in the affected skin) patients, as revealed by plantar biopsies from 10 pachyonychia congenita patients and 10 age/sex-matched controls [106]. Likewise, ζ (OGF) receptor was also expressed in primary human epidermal keratinocytes [107], as well as in the epidermis (basal and suprabasal layers), but not in the dermis of C57BL/6J mice [108]. Moreover, immunoreactivity of Met-enkephalin (OGF) was detected in the basal and suprabasal layers of the epidermis of the animals, but no signals were identified in their dermis [108].

#### 2.1.3. Contradictory Data

In an early study, Salemi et al. failed to identify MOR mRNA and protein expression in the skin of healthy human individuals (females; left deltoid area) but found that DOR and KOR were both present there [109]. Importantly, both DOR and KOR were found to be primarily expressed in dermal fibroblasts and in monomorphonuclear cells. Epidermal keratinocytes appeared to exhibit weak DOR positivity (immunohistochemistry, Western blot), whereas KOR expression was below the detection limit (Western blot) [109]. Moreover, in another study, KOR expression in epidermal keratinocytes could not be confirmed, and the expression of MOR was also very weak, but both receptors were strongly expressed on dorsal root ganglia (DRG) neurons, as well as in the spinal cord of C57BL/6J mice [110].

Likewise, the presence of β-endorphin was demonstrated in the hair matrix, as well as in the sweat gland ducts, but immunoreactivity was absent in the epidermis of healthy volunteers, and this was not changed by UV irradiation exceeding the minimal erythema dose [111]. Importantly, in the manuscript, the authors highlighted two important limitations of their data. First, the antisera they used exhibited a certain degree of cross-reactivity with other POMC-derived peptides, i.e., they may not be entirely specific to β-endorphin. Second, based on the available data, it could not be decided whether this β-endorphin immunoreactivity was due to local production or uptake from the systemic circulation or cutaneous nerves [111].

Although epidermal keratinocytes are capable of degrading Leu-enkephalin (data obtained on isolated keratinocytes of neonatal BALB/c mice) [112], in the early study of Nissen et al., neither Met-enkephalin (OGF) nor Leu-enkephalin were found to be expressed in epidermal keratinocytes or other dermal/epidermal cell types [113], while they could be shown in dermal CD68+ cells (macrophages) [113]. Moreover, the number of Met-enkephalin (OGF)-expressing CD68+ cells was found to be significantly increased in psoriatic lesions as compared to non-lesional as well as healthy skin [113].

Although it is not entirely clear what might underlie the above inconsistencies in the literature, they definitely highlight the necessity of the use of KO-validated (and ideally receptor-splice variant-selective) antibodies, as well as standardized immunolabeling protocols and data reporting methods. The latter would be particularly important to reveal so far overlooked putative interspecies (maybe even interstrain) as well as interindividual, regional and sex-dependent differences in the expression pattern of the members of opioidergic signaling.

### 2.2. Opioidergic Signaling and the Proliferation/Differentiation Balance of Epidermal Keratinocytes—Putative Role in the Development of the Physicochemical Barrier

As mentioned above, appropriate differentiation of epidermal keratinocytes is a key prerequisite of the healthy barrier functions [23]. Importantly, a growing body of evidence suggests that opioidergic signaling is involved in the regulation of the proliferation and differentiation of epidermal keratinocytes.

As mentioned above, similar to its most important (yet unspecific) PENK-derived endogenous ligands, DOR was found to be predominantly expressed in the suprabasal and granular layers of the epidermis [104,105]. This, together with the fact that it was mainly expressed in the cytoplasm of proliferating N/TERT-1 keratinocytes but was translocated to the cell membrane upon Ca^2+^-switch (0.09 mM to 1.2 mM Ca^2+^)-induced differentiation 1 h after the addition of Ca^2+^ [105], suggested that it may be involved in the regulation of the differentiation process of epidermal keratinocytes. In line with this hypothesis, overexpression of DOR markedly suppressed proliferation of said cells (6-day-long culture in monolayer) [105]. This effect was further enhanced by pharmacological activation (SNC80; 100 nM) of DOR. Intriguingly, however, the same SNC80 treatment had no effects on the native (i.e., not DOR overexpressor) N/TERT-1 cells [105].

Moreover, the epidermis of DOR^−/−^ C57BL/6 mice was found to be thinner as compared to the wild-type littermates [114]. Furthermore, the expression of the early differentiation marker K10 [115], as well as levels of K6 (a marker of hyperproliferative keratinocytes [116,117]), and collagen IV were increased in the course of wound healing in a burn wound model of DOR^−/−^ mice. Importantly, in the meantime, the healing process itself appeared to be delayed (a ca. 2-day delay was observed) [114], suggesting that the lack of homeostatic DOR signaling may lead to the initiation of a premature/disturbed differentiation process. Interestingly, DOR overexpression was found to inhibit the proliferation of human keratinocytes, resulting in decreased epidermal thickness in an organotypic human skin equivalent (made of N/TERT-1 cells seeded on rat tail collagen type I and human dermal fibroblasts) [105]. On the other hand, however, it also delayed the upregulation of K10 during in vitro differentiation and decreased its upregulation in the said 3D skin model [105]. This was accompanied by deregulation of involucrin (IVL), loricrin (LOR), and FLG, most likely in an extracellular signal-regulated protein kinase (ERK)-1/2 mitogen-activated protein kinase (MAPK) and POU2F3 (“POU Class 2 Homeobox 3”; a transcription factor involved in the regulation of the epidermal proliferation/differentiation balance [118]) dependent manner [105]. Based on these data, K10 expression appears to be tightly regulated by DOR; lack of homeostatic DOR signaling upregulates, while activation of DOR decreases K10 expression [105,114].

In another study, the activation of DOR by 100 nM Met-enkephalin (OGF) in DOR overexpressor human N/TERT-1 keratinocytes was found to increase the speed of the closure in a scratch assay in a protein kinase C (PKC)-α-mediated manner [119]. These data indicated that DOR destabilizes intercellular adhesion and promotes the migratory keratinocyte phenotype, which is required for fast wound closure [119].

Interestingly, not only DOR^−/−^ but also MOR^−/−^ as well as KOR^−/−^ C57BL/6 mice were shown to have a significantly thinner epidermis (1–2 cell layers instead of 3–4). Moreover, MOR^−/−^ animals exhibited a higher density of epidermal PGP (“protein gene product”) 9.5 positive nerve fibers than the wild-type mice (lack of KOR only tended to increase epidermal nerve density). Importantly, dry skin dermatitis (evoked by twice-a-day 15 s treatment with a mixture of acetone and diethylether (1:1) for 5 days) induced significantly less epidermal hypertrophy in MOR^−/−^ and in KOR^−/−^ animals as compared to the wild-type mice [120]. In line with these findings, a novel, selective [121] KOR agonist (KORA 5a; 200–1000 nM; 48 h treatments) was shown to significantly increase epidermal thickness, as well as the number and proliferation of epidermal keratinocytes in organ-cultured, full-thickness human skin [122]. Surprisingly, it was also found to stimulate apoptosis of epidermal keratinocytes [122].

As mentioned above [119], not only the proliferation/differentiation balance but also migration of epidermal keratinocytes may be influenced by opioidergic signaling. Indeed, 1–10 µM β-endorphin as well as 100 µM morphine, but not 10 µM dynorphin (its exact isoform was not revealed in the original paper), promoted migration of primary human keratinocytes isolated from neonatal foreskin [123]. The effect of β-endorphin could be inhibited by 100 µM naltrexone [123]. Similarly, the DOR-preferring (Figure 2) PENK-derivative Leu-enkephalin (20 µM) promoted migration of HaCaT keratinocytes in the scratch wound assay. The authors concluded that the actions were mediated in a DOR-dependent manner, since co-administration of naltrindole (10 µM) could prevent the effect [124]. However, in the said concentration, naltrindole can also block MOR and KOR (Figure 1). The cellular effects of Leu-enkephalin were mediated via the activation of the ERK1/2 → pP90^RSK^ cascade and involved the upregulation of matrix metalloproteinase (MMP)-2 and -9 expression (24 h treatments) as well as the disassembly of hemidesmosomes [124]. Intriguingly, the administration of β-neoendorphin (a KOR-selective endogenous opioid; Figure 2) yielded very similar results. Indeed, β-neoendorphin was found to accelerate migration of HaCaT keratinocytes (10–20 µM; 20 h) via the activation of ERK1/2 → pP90^RSK^ signaling and to upregulate MMP-2 and -9 mRNA expression (24 h). Thus, at their given test concentrations (~10–20 μM), Leu-enkephalin and β-neoendorphin appeared to activate highly convergent, overlapping (perhaps identical) signaling pathways in human keratinocytes. The wound-healing effect was mainly mediated through the acceleration of keratinocyte migration without affecting cell proliferation [125]. Moreover, β-neoendorphin was also capable of increasing the migration of mouse embryonic fibroblasts (12 h) [125].

MOR and β-endorphin protein expression was found to transiently increase in the epidermis in the course of the wound-healing process in Wistar rat with deep partial-thickness scald [126]. Likewise, mRNA expression of MOR, DOR, and KOR was elevated in hypertrophic scars as compared to normal skin in situ, as well as in the cultured keratinocytes and fibroblasts isolated from the said samples [127]. β-endorphin (60–80 nM; 48 h treatments) stimulated K16 expression in the suprabasal layers. Moreover, it downregulated MOR expression (20–156 nM; 48 h treatments) in organ-cultured human epidermis obtained by dermatome (thickness ~0.5 mm; d = 4 mm) from the upper leg of the donors in a most likely MOR-dependent manner, since naltrexone (80 nM) and a polyclonal guinea pig anti-MOR antibody (80 nM) could prevent the action of 60 nM β-endorphin [128]. Importantly, the authors also showed that MOR is downregulated, whereas K16 is upregulated in psoriatic lesional skin compared to the peri-lesional symptom-free skin [128].

Next, in the same organ-culture model, the transforming growth factor (TGF)-β2 receptor as well as K16 were found to be concentration dependently upregulated in the epidermis by β-endorphin (16–125 nM; 48 h treatments). The effect was, again, MOR dependent, since 80 nM naltrexone or a polyclonal guinea pig anti-MOR antibody (80 nM) could prevent the action of 60 nM β-endorphin on TGF-β2 receptor. Moreover, the expression of MOR on keratinocytes close to the wound margin was downregulated in chronic wounds (ulcus cruris), but, interestingly, hardly any downregulation was noted in acute wounds (erosion and burns) [129].

The differentiation of primary human epidermal keratinocytes (as monitored by assessing transglutaminase (TGase) activity and keratin 10 (K10) expression) was suppressed by Leu-enkephalin (10–1000 nM), Met-enkephalin (10–1000 nM), and DADLE (“D-ala_5_-D-leu_5_-enkephalin”, a synthetic agonist of human MOR and DOR; 1–1000 nM) but not by β-endorphin (0.1 pM–1 µM). Importantly, 30 min pre-incubation with 100 nM naltrexone only prevented the effects of Met-enkephalin and Leu-enkephalin, but not of DADLE, on the TGase activity [130]. Furthermore, 1 h treatment with 1 µM Met-enkephalin suppressed the proliferation of keratinocytes by 15–20% as demonstrated by ^3^H-thymidine incorporation assay [130]. Considering the target spectrum of Met-enkephalin (Figure 2), one may speculate that this latter effect might have been due to the activation of ζ (OGF) receptor that was shown to suppress proliferation of several cell types, including epidermal keratinocytes [107,108,131]. Met-enkephalin, on the other hand, was also shown to promote outgrowth of murine keratinocytes from skin explants [132]. Indeed, according to a recent animal study conducted on C57BL/6J (B6) mice, CD25+/Foxp3+ T_reg_ cells were expanded by UVB exposure (500 mJ/cm^2^; i.e., 2× minimal erythema dose in the given model system), and these cells expressed PENK at a much higher level as compared to the CD25−/CD4+ “conventional” T cells [132]. Importantly, the PENK-derived Met-enkephalin (500 nM; 2 days) promoted the outgrowth of epidermal keratinocytes in an ex vivo skin explant assay, and a priori UVB exposure promoted wound healing in an in vivo wound closure assay in a PENK+ T_reg_-dependent manner [132].

Interestingly, a slightly higher dose (1 µM) of Met-enkephalin (OGF) suppressed the proliferation of primary human epidermal keratinocytes (72–96 h) in another study. The effect was mediated in a ζ (OGF)-receptor-dependent manner, since both naltrexone- (1 µM) and siRNA-transfection-mediated selective gene silencing of ζ (OGF) receptor could abrogate it. Moreover, the above naltrexone treatment as well as the silencing of ζ (OGF) receptor enhanced proliferation of the keratinocytes [107]. The anti-proliferative effect of Met-enkephalin (OGF) was most likely related to the activation of p16^INK4a^ (also known as cyclin-dependent kinase inhibitor 2A, a known tumor suppressor) and p21^WAF1/CIP1^ (also known as cyclin-dependent kinase inhibitor 1) [107]. Likewise, the administration of Met-enkephalin (OGF; 10 mg/kg; i.p.) 4 h prior to sacrificing the animals suppressed epidermal proliferation in the tail skin of C57BL/6J mice 24 h after tape stripping, while naltrexone (20 mg/kg; i.p.) increased it as compared to the PBS-treated control animals [108]. Intriguingly, naloxone (10 mg/kg; i.p.) did not increase the proliferation but almost completely abrogated the effect of Met-enkephalin (OGF) [108]. Last, but not least, Met-enkephalin (OGF) also suppressed the proliferation of A431 cells (a human squamous cell-cancer-derived cell line) by activating OGF receptor [131].

Intriguingly, however, certain pieces of evidence suggest that the anti-proliferative effect Met-enkephalin (OGF) might even be context dependent. Indeed, repeated intradermal injection of Met-enkephalin (OGF; 45 nmol in 50 μL at 0, 24, and 48 h in the forearm of healthy volunteers) appeared to increase epidermal proliferation, as monitored by the determination of the number of Ki67+ cells [133]. However, in the said study, no statistical analysis was performed to assess the significance of the differences [133].

Finally, it should also be noted that PENK (precursor of Met-enkephalin (OGF) as well as Leu-enkephalin; Figure 2) levels were found to be significantly increased both in serum and in psoriatic lesions in patients compared with healthy controls. PENK levels were significantly higher in the lesional skin of the patients as compared to the non-lesional skin, but no significant correlation was found between PENK levels and patient age, disease duration, or disease severity (monitored by Psoriasis Area and Severity Index) [134]. This seems to be particularly interesting, since in another study, 12 psoriatic patients were treated topically with calcipotriol ointment (50 µg/g twice a day), mometasone furoate ointment (0.1%), or appropriate vehicles for 14 days. As expected, both treatments improved the clinical symptoms and the histological findings (reduction in epidermal thickness as well as in the degree of parakeratosis; minor improvement in the number of CD3+ and CD68+ cells) [135]. Moreover, the level of cutaneous Met-enkephalin (OGF) was significantly decreased by both treatments [135]. This suggests that upregulation of Met-enkephalin (OGF) in the lesional epidermis may be a failed attempt of the body to suppress hyperproliferation of epidermal keratinocytes.

### 2.3. Opioidergic Signaling and the Cutaneous Immune System—Potentially AD-Relevant Aspects

#### 2.3.1. Cutaneous Opioidergic Signaling Is Involved in Mediating Local Immune Responses

Opioidergic signaling was shown to deeply influence the immune responses [68,136], and certain elements of the innate immune system were also shown to regulate opioidergic signaling. Indeed, the expression of PENK was upregulated in primary human epidermal keratinocytes by the toll-like receptor (TLR)-2 and TLR4 activator PAM3CSK4 and lipopolysaccharide (LPS), respectively (both applied at 1 µg/mL for 1 h), as well as by UVB irradiation (25 mJ/cm^2^; after 1 h incubation) [104]. Unfortunately, however, further processing of PENK was not investigated; therefore, it remains to be elucidated in future studies whether the levels of the active derivatives (e.g., Met-enkephalin or Leu-enkephalin) are also changed under these pro-inflammatory conditions.

Intriguingly, neither UVA (20 mJ/cm^2^), nor UVB (20 mJ/cm^2^), nor their combination influenced the immunoreactivity of β-endorphin (or β-endorphin-like peptides) in neonatal-foreskin-derived human keratinocytes (immunocytochemistry) [98]. However, a more recent study suggests that UV (50 mJ/cm^2^ of UVB 5 days per week for 6 weeks) irradiation-induced elevation of plasma β-endorphin levels in C57BL/6 mice may be the consequence of the elevated β-endorphin synthesis in epidermal keratinocytes [137]. Importantly, the said increase (from ca. 200 pg/mL to ca. 300 pg/mL) in the β-endorphin level was proven to be biologically relevant, since it increased the pain thresholds, as determined by mechanical and thermal nociception. Moreover, the effect could be reversed by naloxone (10 mg/kg i.p.; 15 min prior to the respective nociceptive tests) [137]. These UV-induced nociceptive and behavioral effects were absent in β-endorphin^−/−^ mice and were shown to be coupled with the p53-mediated upregulation of POMC in epidermal keratinocytes [137]. Interestingly, blue light (λ = 453 nm; 20–40 J/cm^2^) irradiation could also induce β-endorphin release from cultured human epidermal keratinocytes (as measured 1–4 h after irradiation), as well as from human skin (40 mW/cm^2^ from 50 cm; total dose: 72 J/cm^2^ within 30 min), and increased systemic β-endorphin levels in humans in vivo similar to UVB irradiation, most likely via stimulating nitric oxide production of the keratinocytes [138]. Considering that excessive sunlight exposure is well known to worsen the symptoms of AD patients [18] and that serum β-endorphin level was found to be elevated in some [139,140] (but, intriguingly, not all [141]) AD patients, these data may suggest that dysregulation of cutaneous β-endorphin signaling may contribute to the development of the symptoms of AD (see later). Last, but not least, β-endorphin was also upregulated in 12-O-tetradecanoylphorbol-13-acetate (TPA; also known as phorbol 12-myristate 13-acetate (PMA))-painted skin (8.1 nM administered in 100 μL acetone for 24 h) of BALB/c mice, i.e., in a model for irritant contact dermatitis [142].

Importantly, although certain pieces of evidence argue that β-endorphin may context dependently promote immune responses, many findings support the concept that it usually dampens them (for details, see Ref [143]), while other data show that it exerts a complex immunomodulatory effect. For example, β-endorphin (10 nM) increased the LPS (1 µg/mL)-induced secretion of both IL-1β (pro-inflammatory cytokine) and IL-10 (anti-inflammatory cytokine) but suppressed the release of tumor necrosis factor (TNF)-α (pro-inflammatory cytokine) in the course of 24 h treatments on XS52 dendritic cells (a murine model of human Langerhans cells (LCs) originated from the epidermis of newborn BALB/c mice) [144]. Similar effects were seen at the mRNA level on freshly isolated murine LCs as well [144]. Thus, the biological effects of the elevation of β-endorphin production in the above inflammatory conditions are most likely context dependent and may be influenced by other factors, e.g., alterations in the local expression pattern of its receptors. From this perspective, it seems to be important to emphasize that 24 h treatment with the highly AD-relevant [145,146] cytokine IL-13 (100 ng) upregulated MOR (the most important receptor of β-endorphin) in human monocyte-derived LCs that were activated by a 48 h treatment of the combination of 1 µg/mL LPS and poly-(I:C) (10 µg/50,000 cells) [147]. Moreover, MOR expression was detected in all epidermal layers, and its intensity was not significantly different between healthy individuals and AD patients. However, it was significantly reduced by PUVA (psoralen-ultraviolet A) therapy (0.6 mg/kg 8-methoxypsoralen p.o., 2–6 J/cm^2^) of the said patients [103]. Thus, although the effect of IL-13 on the MOR expression of the keratinocytes has not been investigated yet, based on the above findings [103], one might speculate that IL-13-mediated pathological inflammatory response may prevent activation-induced downregulation of MOR [99,128] in lesional keratinocytes of AD patients, maintaining pathologically prolonged MOR activity.

Finally, it should also be mentioned that PK20 (H-Dmt-D-Lys-Phe-Phe-Lys-Lys-Pro-Phe-Tle-Leu-OH; a synthetic opioid–neurotensin hybrid peptide encompassing endomorphin-2 analog and modified fragment of neurotensin (8–13) activating both MOR and DOR [82]) was found to decrease ear thickness and inflammatory cell infiltrate in a dinitrofluorobenzene (DNFB)-induced contact hypersensitivity model (topical treatments on 7–8-week-old male BALB/c mice). Indeed, PK20 was administered in a neutral hydrophilic cream on both sides of the left ear (250 μg PK20 per ear) of the animals 2 h after the DNFB challenge, whereas the right ear received an equal amount of vehicle. Importantly, PK20 treatment significantly decreased concentrations of TNF-α, monocyte chemoattractant protein 1 (MCP-1, also known as C-C motif chemokine ligand 2 (CCL2)), and IL-1α, while it only tended to suppress the levels of IL-1β and KC (also known as CXCL1, a potent chemoattractant of neutrophils [148]) in tissue homogenates derived from equal pieces of the ears [149]. Moreover, the level of thiobarbituric acid-reactive substances (i.e., indicators of lipid peroxidation) was also found to be significantly decreased [149]. Although selective antagonists and/or MOR^−/−^ or DOR^−/−^ animals were not used in this study (i.e., it is unclear whether MOR and DOR were indeed involved in mediating the effects of PK20), these data still argue that topical opioid receptor agonists may exert beneficial effecs in certain types of cutaneous inflammatory conditions.

#### 2.3.2. As Compared to MOR, KOR and DOR Are More Likely to Mediate Local Anti-Inflammatory Actions in the Skin

Putative anti-inflammatory potential of the cutaneous opioidergic signaling was further demonstrated in other studies. Indeed, acute morphine administration (0.1–10 mg/kg, s.c.) was found to reduce peri-incisional production of IL-1β, IL-6, TNF-α, granulocyte colony-stimulating factor (G-CSF), and KC (also known as CXCL1) in the plantar skin of 12–14-week-old male C57BL/6J mice [150]. The higher morphine concentration could also reduce myeloperoxidase activity as well as neutrophil accumulation [150]. Likewise, KOR (U50488; 7.5–60 µg) and DOR (DPDPE; 30–60 µg) agonists decreased the cutaneous ROS-induced inflammatory response (monitored by edema formation-induced swelling), which was evoked by the intradermal co-injection of 2 IU glucose oxidase in male Wistar rats [151]. The effect of DPDPE was partially reversed by the co-injection of 120 µg naltrexone [151]. Intriguingly, however, the MOR agonist (and partial KOR agonist) DAMGO (also known as Tyr-D-Ala-Gly-(NMe)Phe-Gly-ol; 7.5–60 µg) had no effect [151], suggesting that activation of MOR alone may not be enough to evoke the anti-inflammatory action, and/or KOR agonism plays a key role in it. Last, but not least, it is also important to note that none of the compounds was effective when applied intraperitoneally [151], arguing that the anti-inflammatory action was most likely dependent on local cutaneous effects.

The idea that KOR may be responsible for the aforementioned cutaneous anti-inflammatory effects of certain opioids was further supported by other studies. In a DNFB-induced contact dermatitis model of male Swiss Webster mice, the KOR agonist, but DOR and MOR partial agonist (Figure 1), Nalbuphine (10 mg/kg; s.c.; 20 min prior to DNFB), was found to attenuate pruritus, as well as to decrease IL-31 and to increase IL-10 levels in the skin of the animals. Importantly, these effects developed without decreasing locomotion (i.e., no sedative effect was observed). Interestingly, however, both 3 and 10 mg/kg Nalbuphine (s.c.) resulted in anhedonic behavior, since they moderately decreased self-grooming in the splash test (i.e., spraying 0.7 mL of 10% sucrose solution on the flank of the mice 1 h after Nalbuphine administration) [152].

In another study, novel selective KOR agonists were found to exert potent anti-inflammatory actions in vivo following topical application in acute (5 mg arachidonic acid in 20 µL acetone topically) and oxazolone-induced chronic cutaneous inflammation in male ICR mice (arachidonic acid) and male BALB/c mice (oxazolone) [121]. Likewise, topically applied nalfurafine (0.2–1% solutions dissolved in dimethyl sulfoxide (DMSO)) was found to exhibit anti-inflammatory and anti-pruritic effects in the oxazolone-induced model of AD in BALB/c mice [153]. Inflammatory response was induced by treating the right ear of each mouse by oxazolone (1% solution) on days 0, 7, 9, and 11, and then the same ear was treated by the said doses of nalfurafine on days 11–18. Importantly, both doses of nalfurafine reduced ear thickness as well as the number of scratch events, and, intriguingly, they also induced a transient (lasting for 3–4 h after the administration) decrease in the general activity of the mice on day 11, but not on day 18 [153]. Moreover, 1% nalfurafine also decreased dermal infiltration of CD4+ and CD8+ cells on day 19 [153].

Furthermore, in a canine model of AD, 14 beagles were challenged with house dust mites every 3–4 days for a total of 9 challenges to induce an AD-like phenotype. The animals were treated by the topical KOR agonist asimadoline (0.6 mL of 1% gel to the inguinal region twice daily for three weeks starting on day 7) or placebo in a prospective cross-over study (i.e., after a 4-week washout period, dogs were crossed-over, the study was repeated, and the results were analyzed using combined data). Importantly, by the end of the experiment (day 28), asimadoline significantly improved the Canine Atopic Dermatitis Extent and Severity Index (CADESI) as compared to the placebo control. Interestingly, however, no improvement in the pruritus score was noted [154].

Besides the above findings, preliminary in vitro data also argue that activation of the KOR on epidermal keratinocytes may exert anti-inflammatory actions. Indeed, nalfurafine (10 nM) was found to suppress the pro-inflammatory effects of the TLR3 activator poly-(I:C) on HaCaT keratinocytes (24 h treatments) in a KOR-dependent manner, since co-administration of nor-binaltorphimine (nor-BNI; 100 nM) prevented the action [155]. Likewise, another selective KOR agonist (WOL071-007) alleviated imiquimod-induced psoriasis-like inflammation in BALB/c mice in a KOR-dependent manner (co-administration of nor-BNI prevented the effect) with a similar efficiency to anti-TNF-α antibody [156]. Finally, both the peripherally restricted KOR agonist asimadoline, as well as the peripherally non-restricted WOL071-007, could suppress ear swelling in an arachidonic-acid-induced murine model of inflammation [157]. (*Note that the above studies, i.e., Refs* [155,156,157], *were only reported in the forms of citable abstracts, thus can only be considered as preliminary reports.*)

The anti-inflammatory effects of KOR are most likely not restricted to actions on epidermal keratinocytes. Indeed, α-neoendorphin, an endogenous decapeptide (N-YGGFLRKYPK-C; 5 µg/mL) that preferentially activates KOR (Figure 2) was shown to reduce UVB (35 mJ/cm^2^)-induced skin photoaging (monitored by elevated ROS production, increased MMP-2 and -9 expression and collagenase activity, reduced procollagen expression) by activating autophagy in the human dermal fibroblast cell line Hs68. The effects were mediated via a mammalian target of rapamycin (mTOR)-Beclin-1-coupled signaling pathway [158]. Importantly, however, the overall picture might be even more complex, since the MOR/KOR/DOR (and at high doses, OGF receptor) antagonist naltrexone was also found to be beneficial in alleviating pruritus as well as in treating chronic cutaneous inflammatory conditions [159].

Last, but not least, it should also be noted that 50 µL of Met-enkephalin (OGF; 16, 30, and 45 nmol) induced a time- and dose-dependent flare reaction (maximal reaction: after 1 min; disappearance: after 45 min) when injected intradermally once in the forearm of healthy volunteers, while no wheal reaction was observed in the course of the 120 min observation period. Flare reaction was partially inhibited by cetirizine pretreatment (an antihistamine; 10 mg administered orally 1 h prior to the test) [133]. In normal skin, Met-enkephalin (OGF) seemed to increase the number of CD3+ (T lymphocytes) as well as CD68+ cells (macrophages), while in a tuberculin-induced delayed-type hypersensitivity reaction, the same treatment appeared to reduce the tuberculin-induced infiltration of the CD3+ and CD68+ cells [133]. However, statistical analyses of the alterations in the number of CD3+ and CD68+ cells were not performed [133].

#### 2.3.3. Effects of the Opioidergic Signaling on Mast Cells (MCs)

Considering that mast cells (MCs) are key effector immune cells involved in atopic diseases (including AD) and that they are central players in the development of chronic pruritus characteristic of AD [45,160,161], it is worth to briefly summarize how opioids influence MC activity.

The first notion that MCs may be regulated by opioidergic signaling dates back to 1984. In that study, it was shown that intradermal injection of several opioids (codeine, morphine, and meperidine; doses: 16.7 pmol to 181.9 nmol) induced MC degranulation in humans [162]. Moreover, other test compounds also induced wheal-and-flare reactions with the following order of potency: dynorphin > β-endorphin > (D-Ala,^2^-D-Leu^3^)-enkephalin (DADLE) ~ morphiceptin (a β-casomorphin-derived MOR agonist tetrapeptide; sequence: Tyr-Pro-Phe-Pro-NH_2_) at dose ranges of 0.3 to 8.45 nmol [162]. Co-administration of naloxone (12.22 nmol) could decrease the effect of morphine (1.05 and 3.5 nmol) but not of dynorphin (0.06–3.12 nmol) or histamine (200 ng), suggesting that different opioids exerted their effects through the activation of different receptors and cellular targets [162].

In another study, intradermally injected 20 µL fentanyl and morphine solutions (the latter being more efficacious) produced concentration-dependent wheal-and-flare responses in human volunteers (5 µM to 1.5 mM). Importantly, the co-administration of naloxone (500 µM) almost completely abrogated the effect of fentanyl, whereas it only slightly (but still significantly) suppressed the action of morphine [163], further arguing that opioid-induced wheal-and-flare responses may develop via the activation of differential signaling pathways.

Although the above early studies suggested that DOR and/or KOR agonism may also activate MCs, more recent data argue that the overall picture is more complex. Indeed, KORA 5a (200 nM) significantly decreased the number of c-Kit-positive MCs, but it did not significantly alter the number or degranulation of mature (tryptase- or toluidine blue-positive) MCs [122], making KOR an unlikely candidate for positive regulation of MC activity.

In another study, the effects of selected opioids (codeine, meperidine, fentanyl, alfentanil, sufentanil, remifentanil, and buprenorphine) as well as naloxone were investigated on MCs using intradermal microdialysis (flow rate: 4 µL/min) on 60 healthy human subjects. Importantly, only codeine (0.5 mg/mL), meperidine (1 mg/mL), and morphine (used as positive control at 0.5 mg/mL) induced MC activation with the release of tryptase and histamine, leading to protein extravasation, flare reactions, and itch sensations. Because the co-injection of naloxone (0.2 mg/mL) only tended to attenuate (morphine) or did not influence (the other two cases) these effects, the authors concluded that MOR is most likely not involved in the activation of MCs in this experimental setup [164].

Likewise, although the supposedly MOR-selective agonist (Figure 1) codeine induced degranulation of skin MCs in situ (EC_50_: 29.5 μM), the effect could not be prevented by naloxone pretreatment (1–100 μM; applied 15 min prior to codeine), making MOR an unlikely candidate in mediating the effect. Indeed, codeine was found to exert its action via the (direct or indirect) activation of MAS-related GPR family member X2 (MRGPRX2) receptor that was followed by the β-arrestin-1-mediated internalization and downregulation of the receptor, leading to desensitization toward other degranulation signals (e.g., compound 48/80) [165].

Finally, it should also be mentioned that, according to some data, NOP receptors may also be involved in regulating cutaneous MC function. Indeed, the intradermal administration of nociceptin (5–5000 pmol) increased vascular permeability in a dose-dependent manner in male Wistar rats, and the effect could be prevented by the co-administration of the H_1_ receptor blocker pyrilamine (5 nmol), arguing that MC activation might be involved in the process [166]. The authors also showed that nociceptin induced histamine release from rat peritoneal MCs, and the effect could not be prevented by the co-administration of naloxone (100 µM) but was abrogated by pertussis toxin (an inhibitor of G_i_/G_o_ proteins) pretreatment (1–10 ng/mL 120 min prior to nociceptin administration), as well as by the addition of Ca^2+^ (0.5–2 mM) [166]. In light of the above data, the authors suggested that the naloxone-insensitive, G_i_-protein-coupled NOP receptors may mediate the effects of nociceptin in this model system [166].

#### 2.3.4. Opioidergic Signaling May Be Involved in Regulating the Balance between T_h_1- and T_h_2-Type Immune Response

As mentioned above, a T_h_2/T_h_22-dominated immune response is characteristic of AD [1,2]. Importantly, this imbalance in the immune system (especially during early ages) appears to exert profound effects on the health of the patients that extend beyond the (already very unpleasant) cutaneous symptoms of AD. Indeed, AD-coupled early life T_h_2 overload may increase the risk of developmental and psychological disorders, including e.g., autism spectrum disorder, memory impairment, speech disorders, or even certain forms of epilepsy [167]. Thus, normalization of the T_h_2-dominated immune phenotype may have additional benefits even beyond improving the symptoms of AD. Importantly, among others, opioidergic signaling is involved in regulating the balance between T_h_1- and T_h_2-type immune responses.

Acute and chronic treatment with naloxone (5 mg/kg; s.c.) suppressed IL-4- and increased IL-2- and interferon (IFN)-γ-release in splenocytes isolated from normal BALB/cJ and C57BL/6 male mice following immunization with keyhole limpet hemocyanin (KLH) protein antigen (100 µg in 200 µL saline; i.p.) [168]. In light of these data, opioid receptors (or at least one of them) may play a role in shifting the immune response in a T_h_2-direction [168]. In line with this observation, naloxone (5 mg/kg; s.c.; twice a day, started on the day of the transplantation) also accelerated the rejection of skin grafts in 7–8-week-old female C57BL/6N and C3H/HeN mice [168]. Moreover, it suppressed anti-KHL IgG production in BALB/cJ mice but had only negligible effects on the anti-KHL IgM level [169].

The promotion of a shift toward a T_h_2-type immune response by the opioid receptor agonist morphine was also demonstrated by multiple other studies [170,171]. When testing anti-CD3/anti-CD28-stimulated peripheral blood monomorphonuclear cells (PBMCs) of human subjects as well as splenocytes of wild-type and MOR^−/−^ CB6F1/J mice, morphine (10–100 ng/mL; 96 h) decreased IL-2 and IFN-γ while promoting IL-4 and IL-5 production of human PBMCs, and similar effects were seen in the splenocytes of the wild-type mice. However, the effect was completely missing in MOR^−/−^ animals, suggesting that activation of MOR is crucial in the development of the T_h_2-type immune response upon the said morphine treatment [170].

In another study, the same group found that morphine (100–500 ng/mL; 72 h) increased cAMP levels of anti-CD3/anti-CD28-induced splenocytes of C57/S129 mice [171]. Further investigation revealed that morphine (100–200 ng/mL; 20 min) activated the ERK1/2, p38, as well as the cAMP response element-binding protein (CREB) pathways [171]. Moreover, its effects on the IL-4 release could be prevented by the inhibition of p38 (0.1–1 µM SB203580) but not of protein kinase A (PKA; 0.5–1 µM H89, or 1 µM myristoylated PKI [14,15,16,17,18,19,20,21,22] amide), or ERK1/2 (10–50 µM PD98059) [171].

Intriguingly, however, to make matters more complex, an investigation of triple KO (i.e., MOR^−/−^/KOR^−/−^/DOR^−/−^) animals (maintained in a hybrid 50% 129/SvPas: 50% C57BL/6J genetic background) revealed that they exhibited a T_h_2-directed shift in their immune system. This suggests that global endogenous opioid tone (perhaps through KOR and/or DOR) drives T lymphocytes toward a T_h_1 profile under physiological conditions [172].

### 2.4. Opioids in the Skin—Microbiota Communication

Dysregulation of the cutaneous microbiota, and especially the loss of homeostatic “biodiversity”, leading to the overgrowth of pathogenic *Staphylococcus aureus* or *Candida albicans* strains, appears to be an important factor in the pathogenesis of AD [30,31,32,173]. Although these changes most likely do not represent the root cause of AD, the colonization of pathogenic *Staphylococcus aureus* strains undoubtedly contributes to the development and worsening of AD lesions via, among others, an EV-mediated interspecies communication [63,174].

From this perspective, it is fascinating that opioidergic signaling may be involved in skin–microbiota communication. Indeed, β-endorphin exposure (100 nM; 4 days) was found to increase phospholipase activity (a virulence factor in several yeasts, including e.g., *Candida albicans*) in *Malassezia* species isolated from seborrheic dermatitis lesional skin (but, interestingly, not in the ones isolated from healthy skin) [175]. Thus, considering that the composition of the skin fungal microbiota also exhibits characteristic alterations in AD (similar to what is documented for bacterial flora) [30], further studies are encouraged to investigate whether and how cutaneous β-endorphin production impacts the biodiversity of skin microbes, possibly favoring the growth of certain pathogenic strains over homeostatic ones.

Importantly, not only β-endorphin, but also Met-enkephalin (OGF), may be involved in the host–microbiota communication. Indeed, 1 μM Met-enkephalin (OGF) was also shown to slightly, but significantly, suppress growth of certain bacterial strains (*Staphylococcus aureus* (ATCC 25923), *Pseudomonas aeruginosa* (ATCC 27853), and *Serratia marcesans*), whereas naltrexone (1 μM) was found to increase it in the course of 7 h treatments [176]. Interestingly, the authors also demonstrated that Met-enkephalin (OGF) can be produced by bacteria, and it also has a saturable binding site in *Staphylococcus aureus* (K_d_: 1.8 ± 0.2 nM; binding capacity: 202 ± 27 fmol/mg protein), i.e., it appears to be an autocrine/paracrine regulator of (at least certain) members of the microbiota [176].

Moreover, morphine (known targets: MOR >> KOR > DOR [177]) and meperidine (known targets: MOR > KOR > DOR [178]), but not fentanyl (known targets: MOR >> KOR > DOR [177]), exhibited direct antibacterial effects. They were found to be effective against several strains of coagulase negative as well as positive *Staphylococci*, *Escherichia coli*, *Klebsiella enterobacter*, and *Pseudomonas aeruginosa* (minimal inhibitory concentration (MIC) of meperidine: 6.25 mg/mL; MIC of morphine: 20 mg/mL for *Pseudomonas aeruginosa* and 10 mg/mL for other bacteria) but, intriguingly, they were not efficient against *Proteus* species [179]. Furthermore, the non-opioid local anesthetic Bupivacaine (5 mg/mL) also suppressed growth of several microbes (*Staphylococcus aureus* (ATCC 25923), multi-resistant as well as sensitive *Staphylococcus epidermidis*, *Streptococcus pneumoniae*, *Streptococcus pyogenes* (A), *Streptococcus faecalis*, *Bacillus cereus*, *Escherichia coli* (ATCC 25922), and *Candida albicans*), but it had no effect against *Pseudomonas aeruginosa* (ATCC 27853) [180]. Importantly, morphine at 2 mg/mL was found to be ineffective [180], which is not surprising in light of the previous study [179]. Altogether, these findings raise the question whether, similar to several phytocannabinoids [181], plant-derived opioids and opioid derivatives also exert overlooked, but clinically potentially relevant, antimicrobial effects.

The mechanism of the antimicrobial activity remains to be elucidated in future studies, and it is unknown whether resistance can develop against it. However, the fact that certain widely used opioids exert relevant antibacterial activity against *Staphylococcus* and *Candida albicans* strains raises the possibility that they could be used as adjuvant treatment of AD-accompanying cutaneous dysbiosis (obviously only in appropriate topical formulations that could prevent their systemic adverse effects). However, in the course of clinical investigation of such opioid-containing formulations, special caution will be needed, since, according to a recent case report, certain opioids (thebaine, morphine, norhydroxymorphinone, codein, and oripavine) can cause occupational allergic contact dermatitis [182]. Finally, the putative role of endogenous opioids (not only the already investigated β-endorphin and Met-enkephalin (OGF), but all the other keratinocyte-derived endogenous opioids as well) in shaping cutaneous microbiota should also be investigated.

### 2.5. Opioidergic Signaling and Itch

Itch, i.e., the “unpleasant sensation that elicits the desire or reflex to scratch” (definition of the late German physician Samuel Hafenreffer), is a complex sensory phenomenon that is known to be regulated by a number of signaling pathways [183,184,185,186,187], and it is an important symptom that severely impairs the quality of life of AD patients [188,189]. However, itch is much more than a mere symptom of the disease. Indeed, the “itch–scratch cycle” and the subsequently developing scratch injuries were shown to contribute to the pathogenesis of AD (and also of psoriasis) [3,21,46,47].

It is now obvious that pruritogen release from epidermal keratinocytes [190] and MCs [161], as well as the disturbance of opioidergic signaling, can contribute to the development of itch [183,184,185,186,187,191,192,193]. Thus, it is not surprising that pruritus is one of the most common side effects of opioid use. In fact, 2–10% of chronic oral opioid users (both used-as-prescribed and misused) suffer from itch, which is usually a generalized, antihistamine unresponsive pruritus [193].

Importantly, depending on the targeted receptors and certain other factors, opioids can exert both pro- and antipruritic effects. Indeed, the activation of MOR increases, whereas KOR agonism usually (but not always; see below) alleviates itch, introducing KOR agonists (e.g., nalfurafine [194]) as well as certain antagonists that primarily target MOR (e.g., naltrexone [177]) as promising, novel antipruritic agents [159,195,196,197,198,199,200,201,202].

#### 2.5.1. Effects of MOR Antagonism and/or KOR Agonism on Pruritus—Animal Studies

As mentioned above, the pro-pruritic effect of MOR agonism was proven in several studies. For example, scratching behavior was investigated in 2–4-month-old male C57BL/6 mice in response to the intradermal injection of the synthetic MOR agonist (and KOR partial agonist; Figure 1) 2-Ala-4-mephe-5-gly-enkephalin (DAMGO; 50 µL (nape) or 20 µL (cheek) of a 200 µM solution; i.d.). Scratching was quantified in MC-deficient (Kit-Wsh/Wsh), as well as in protease-activated receptor 2 deficient (PAR-2^−/−^), and in transient receptor potential vanilloid 1 deficient (TRPV1^−/−^) mice or following the resiniferatoxin (RTX)-mediated ablation of TRPV1+ sensory neurons in wild-type animals. The above DAMGO treatment elicited scratching behavior in a most likely MOR-dependent manner, since it could be abrogated by the peripherally restricted MOR antagonist naloxone-methiodide (1 mg/kg; s.c.; 30 min prior to DAMGO). Importantly, DAMGO-induced itch responses were not altered in MC-deficient, PAR-2^−/−^, or TRPV1^−/−^ mice as compared to their respective wild-type controls [203]. Because ablation of TRPV1+ neurons as well as of acute TRPV1 activation by capsaicin (0.05%; i.d.) abrogated DAMGO-induced itch, while allyl isothiocyanate (AITC, a transient receptor potential ankyrin 1 (TRPA1) agonist; 0.075%; i.d.) pretreatment had no effect, the authors concluded that peripheral DAMGO-induced itch was dependent on the presence of TRPV1-expressing pruriceptors, but not on TRPV1 itself [203].

Likewise, β-endorphin (100 nmol dissolved in 1 mL sterile water; injected intrathecally) elicited scratch response in rhesus monkeys (*Macaca mulatta*), and the effect could be prevented by the co-administration of dynorphin A (30–100 nmol in the same volume) as well as by naltrexone (10–30 nmol in the same volume) [204]. These data suggested that central activation of MOR exerted a pro-pruritic effect, while KOR agonism alleviated MOR-activation-induced itch.

Naltrexone is a potent antagonist of MOR with excellent oral bioavailability [205]; however, one should also keep in mind that, just like the closely related naloxone, it can antagonize DOR and KOR as well (Figure 1). In an animal study, male C57BL/6J mice were first treated with complete Freund’s adjuvant (CFA) to induce local cutaneous inflammation (intradermal injection), and four days later, alloknesis (i.e., itch evoked by normally non-pruritic touch) as well as several other endpoints were investigated. Importantly, naltrexone (1 mg/kg in 100 μL s.c.; applied 15 min prior to the elicitation of the alloknesis response) significantly reduced alloknesis [206]. In another study, both naloxone (0.3–1 mg/kg, s.c., dissolved in physiological saline solution) and the KOR agonist nalfurafine (0.01–0.05 mg/kg, p.o., dissolved in 5% gum arabic solution) were found to inhibit scratching behavior in ADJM mice (“*atopic dermatitis from Japanese mice*”, also known as “*Traf3ip2/Act 1*-deficient mice”) [207].

The concept that activation of KOR is efficient in alleviating itch was further demonstrated in several other studies. R7bp (“regulator of G protein signaling 7 binding protein”, also known as Rgs7bp) is a palmitoylated membrane anchoring protein expressed in neurons, where it facilitates G_i/_G_o_-directed GTPase activating protein activity [208,209], i.e., it is important in limiting signal transduction via certain G_i_/G_o_-coupled receptors, such as KOR. Importantly, R7bp^−/−^ mice (maintained on a C57BL/6 background) had significantly weaker scratching responses following intradermal (compound 48/80 and chloroquine) or intrathecal (gastrin-releasing peptide) administration of various pruritogens. Moreover, the said pruriceptive defect was not present in KOR^−/−^/R7bp^−/−^ double knockout mice. Furthermore, in a diphenylcyclopropenone-induced model of AD, R7bp^−/−^ mice showed diminished scratching behavior and enhanced sensitivity to the intraperitoneally applied KOR agonist U50488 (0.25 mg/kg), and the effects were most likely coupled with the activation of a KOR+ anti-pruritic neuron population in the spinal cord [208].

Moreover, the KOR agonist (as well as DOR and MOR partial agonist) Nalbuphine (3 and 10 mg/kg; s.c.) prevented TAT-HIV-1 (0.3 mg/kg)-induced itch in C57BL/6J mice. Moreover, Nalbuphine (10 mg/kg; s.c.) was efficient in preventing the pruritic effect of deoxycholic acid (3 mg/kg; s.c.), as well as chloroquine (10 mg/kg; s.c.). The effects were proven to be mediated via the activation of KOR, since they were absent in KOR^−/−^ mice and could be prevented by nor-BNI (a widely used, yet not completely specific (Figure 1) KOR antagonist) pretreatment (20 mg/kg; i.p. 20 h prior to Nalbuphine treatment) [210]. Likewise, as mentioned above, Nalbuphine (10 mg/kg; s.c.; 20 min prior to DNFB) was found to attenuate pruritus in a DNFB-induced contact dermatitis model of male Swiss Webster mice without decreasing locomotion. Importantly, however, as revealed in the splash test, both 3 and 10 mg/kg Nalbuphine (s.c.) resulted in an anhedonic behavior of the animals [152].

Besides, as mentioned above, topically applied nalfurafine (0.2–1% solutions dissolved in DMSO) was found to exhibit anti-inflammatory and anti-pruritic effects in the oxazolone-induced murine model of AD in BALB/c mice, and both doses of nalfurafine reduced the number of scratch events [153]. Importantly, however, they also induced a transient (for 3–4 h after the administration) decrease in the general activity of the mice on day 11, but not on day 18. Thus, the possibility that central behavioral effects might have contributed to the observed reduction in the scratching activity could not be excluded [153].

In another study, subcutaneously injected nalfurafine (10–20 µg/kg) was found to suppress histamine- and chloroquine-induced itch and alloknesis in adult male C57BL/6 mice [211]. Intriguingly, the same dose of nalfurafine was also able to suppress itch, but not alloknesis, in the acetone and diethylether (1:1)-induced dry skin model [211], arguing that the anti-pruritic effect of peripheral KOR agonism may be a context-dependent action.

The concept of such context dependence was further supported by the findings of Takahashi et al. Indeed, topical (0.1–1%) and intraperitoneal (1–10 mg/kg) naloxone, as well as the centrally acting KOR agonist ICI-199441 (1 mg/kg; p.o.), suppressed itch, as monitored by the number of scratching bouts in an imiquimod-induced mouse model of psoriasiform inflammation (8–9-week-old male C57BL/6J mice), and neither ICI-199441 nor topically applied naloxone affected locomotor activity. Importantly, however, the peripherally restricted KOR agonist asimadoline (10 mg/kg p.o.) was ineffective [110]. Here, it has to be noted that the expressions of MOR and KOR were below the detection limit in the epidermis of the animals by Western blot, whereas they could be detected in DRG neurons as well as in the spinal cord. Importantly, imiquimod treatment appeared to increase MOR expression in the epidermis, in DRG neurons, as well as in the spinal cord, while KOR expression remained unaffected [110]. Finally, although topically applied asimadoline (0.6 mL of 1% gel) significantly improved CADESI in a dust mite-induced canine AD model, no improvement in the pruritus score was noted [154]. These findings, together with certain other, preliminary (i.e., published only in the form of a citable abstract) data (comparison of the effects of peripherally restricted and non-restricted KOR agonists), highlight the possibility that anti-pruritic effects of KOR agonism are likely to be coupled with central effects [157].

However, as mentioned above, both KOR and MOR were shown to have several splice variants. One possible explanation for the lack of epidermal signals may be that in the animals used in this study, different splice variants were expressed in the keratinocytes and in the neurons. On the other hand, especially in light of the apparent inefficiency of asimadoline, it is also possible that epidermal MOR and KOR expression is not ubiquitous but may rather depend on the actual model system and/or skin region.

#### 2.5.2. A Deeper Insight into the Role of MOR and KOR in Controlling Pruritus—Itchy Circuits

Research of the last two decades advanced a lot our understanding of pruritus [212]. In the skin, itch is sensed by a group of somatosensory fibers, the cell bodies of which are located in the dorsal root and trigeminal ganglia. Most recently, unbiased single-cell transcriptomic studies identified and characterized heterogeneous subpopulations of pruriceptive neurons in rodents, primates, and humans based on the expression of pruriceptive receptors, related signaling molecules, and itch-specific neurotransmitters, such as natriuretic polypeptide b (NPPB) [213,214,215]. These pruriceptive fibers also express the markers of nociceptive neurons; therefore, they are considered as a subpopulation of the nociceptors, as is established by the selectivity theory of itch [216,217,218]. The pruriceptive fibers project to the spinal cord, where neurons expressing gastrin-releasing peptide (GRP+ neurons) and its synaptic partner, gastrin-releasing peptide receptor (GRPR), play a distinctive role in itch transmission toward higher centers [219,220]. Local spinal circuits regulate itch transmission and provide a substrate for interactions between different sensory pathways [221]. Perhaps the most well-known among these sensory interactions is the inhibition of itch by nociception, as it appears in scratching behavior, i.e., a mechanical counter-stimulation that alleviates itch [222]. In the spinal circuits, inhibitory interneurons originated from Bhlhb5-expressing precursors play a central role in inhibiting the transmission of itch [223,224]. These itch-inhibiting interneurons were found to be dynorphin+, and the systemic application of KOR agonists inhibited the GRPR-mediated itch evoked by intrathecal GRP application. Furthermore, intrathecally applied KOR agonists (U-50,488: 10 μg; nalfurafine: 40 ng) alleviated, but KOR antagonists nor-BNI (1 µg) or 5′-guanidinonaltrindole (5′GNTI, 1 µg) augmented, chloroquine-evoked itch [225].

Importantly, these dynorphin+ inhibitory interneurons displayed tonic activity and were further activated by both nociceptors responsive to noxious chemicals and by menthol-responsive (i.e., TRPM8-expressing cold sensitive) fibers [225]. These data may reveal the cellular substrate of the itch-inhibiting counter-stimulation [226].

KOR, as a target of the antipruritic opioids, was identified in distinct subsets of primary afferents, including some peptidergic nociceptive neurons in both rodents and humans, but interestingly, only ca. 10% of the Mrgpra3+ (“Mas-related G-protein-coupled receptor a3” positive neurons; a potentially pruriceptive subgroup of the sensory neurons) DRG neurons expressed KOR. At any rate, dynorphin (0.1 and 1 µM) inhibited the activity of voltage-gated Ca^2+^ channels in KOR+ DRG neurons, and this inhibition was abolished in the presence of the KOR antagonist nor-BNI (1 µM). Moreover, dynorphin (1 µM) inhibited the release of excitatory neurotransmitters in lamina I, and nalfurafine (20 μg/kg), as well as peripherally restricted KOR agonists ICI204,448 (10 mg/kg), or FE200665 (12 mg/kg), inhibited capsaicin-induced neurogenic inflammation. These results suggest that functional KOR(s) are present both in the central and peripheral terminals of primary sensory neurons. Importantly, same doses of nalfurafine, ICI-204448, and FE200665 (also known as CR665) inhibited chloroquine (200 μg/20 μL, i.d.)-induced itch when the given agonist was applied i.p. 15 min prior to the behavioral testing. These behavioral effects of KOR agonists were not detected in KOR^−/−^ animals [227].

In contrast to the antipruritic effect of KOR agonists, MOR activators are known to induce pruritus. Several lines of evidence suggest that MOR directly activates the pruriceptive pathways in the spinal cord. Indeed, it was found that MOR1D forms heteromers with GRPR in the pruriceptive spinal interneurons, and MOR agonists unidirectionally cross-activate GRPR, resulting in itch in a mouse model (following i.t. administration of morphine (0.3 nmol), DAMGO (0.02 nmol), or fentanyl (unreported dose)) [228]. A molecular interaction between GRPR and the MOR1Y isoform is also suggested in the human spinal cord [229]. However, recent results disputed the excitatory role of MOR agonists (for a detailed review, see Ref [230]). The co-expression of MOR and GRPR was questioned by single-nucleus and single-cell transcriptomic data [231,232], and MOR was proposed to function as an inhibitory receptor in itch-inhibiting inhibitory interneurons, arguing that MOR activation evokes itch through disinhibition, i.e., via liberating the itch pathway from the spinal inhibition [233,234]. Indeed, selective deletion of MOR from inhibitory interneurons diminished its itch-inducing effect without affecting morphine-induced analgesia [234].

Morphine-induced itch may have a peripheral, non-neuronal target as well, but it seems to be independent of opioid receptors. Deletion of MOR in peripheral sensory fibers did not influence morphine-induced itch [234], but morphine (≥10 μM), as well as dynorphin A (1–13) (≥1 μM), were shown to induce degranulation of LAD2 human MCs, most likely via MRGPRX2 [235].

A recent study suggested that an inhibition between itch and pain exists even in the opposite direction, namely, itch may also inhibit pain. It was found that itch-mediating *Grp+* interneurons receive inputs from peripheral nociceptors as well and may transmit pain sensation at moderate stimulation, whereas high-intensity stimulation inhibits the spinal transmission of pain mediated by this neuron population. It was hypothesized that high activation of *Grp*+ neurons activated a pain-inhibiting pathway, thereby suppressing nociception. This anti-nociceptive effect of Grp+ neurons was suspended by naloxone (1 μg or 3.33 nmol) and naltrindol (10 μg or 24.1 nmol), but not by the MOR antagonist peptides CTAP (5 μg or 13.7 nmol) or CTOP (10 μg or 9.43 nmol). Furthermore, the synaptic connection between *Grp*+ and enkephalin-expressing interneurons was shown, and activation of the *Grp+* neurons resulted in enkephalin release. These results argue that itch-mediating *Grp*+ neurons can recruit enkephalinergic interneurons that may be able to inhibit nociceptive pathways via DOR activation [219]. However, recent results disputed that *Grp*+ spinal cord neurons affect nociception. Indeed, it was found that only a minority of the *Grp*+ spinal interneurons received synaptic input from nociceptors [236], and locally restricted manipulation of *Grp*+ neurons in the spinal cord did not reveal any role in nociception. It was hypothesized that the previously observed interactions between nociception and pruriception might be related to *Grp*+ neurons in higher brain centers [237].

#### 2.5.3. Effects of MOR Antagonism and/or KOR Agonism on Pruritus—Human Studies

The stable Met-enkephalin analog FK-33-824 ([D-Ala^2^, MePhe^4^, Met(0)^5^-ol]-enkephalin; 1–10 pmol), as well as β-endorphin (1 pmol), and morphine (10 pmol), were shown to enhance histamine (30 ng)-induced itch and flare responses following intradermal injection in healthy individuals. All compounds were injected intradermally in a final volume of 10 µL, and the effect of FK-33-824 could not be prevented by the co-administration of naloxone (6 nmol; injected 1–2 min prior to the combination of histamine and FK-33-824) or by a 2-day pretreatment with indomethacin (50 mg/day, p.o.). Moreover, prior histamine depletion (100 ng compound 48/80) had no effect either [238].

In an early human study, 25 mg naltrexone or placebo was given orally to 11 symptom-free patients suffering from chronic atopic eczema 60 min prior to the intracutaneous injection of acetylcholine (0.02 mL of 0.55 M solution) and its vehicle (saline (NaCl 0.9%, pH 6.7)) to the other volar forearm [205]. Oral naltrexone reduced the acetylcholine-induced cutaneous flare reaction and suppressed alloknesis (as monitored by measuring the intensity of perifocal itch induced by slight, non-noxious mechanical stimulation around the site of injection) as well [205]. Interestingly, however, the duration and intensity of the itch at the site of acetylcholine injection only tended to be suppressed by naltrexone pretreatment [205].

In another small (n = 15) placebo-controlled, double-blind study, iontophoretically applied histamine-induced focal itch and alloknesis were investigated. The administration of naltrexone (25 mg; p.o. 60 min prior to histamine) was found to significantly reduce both itching and alloknesis in healthy subjects [239]. Moreover, naltrexone could efficiently alleviate pruritus following not only systemic but also topical application. Indeed, a 1% naltrexone-containing cream was efficient in alleviating itch in patients suffering from various pruritic diseases [240]. Importantly, epidermal MOR expression was usually elevated as compared to the initial level in those patients who responded well to the naltrexone treatment, suggesting that topical naltrexone may primarily target MOR at the periphery, in the epidermis. Furthermore, the authors made some other intriguing observations, namely: (i) topical application of naltrexone had much less effect if the site of pruritus was the trunk rather than the extremities; (ii) naltrexone had less or no effect in the case of nephrogenic pruritus but was highly efficient in AD, which was further confirmed in a double-blind, placebo-controlled trial involving 40 patients [240]. This suggests that there might indeed be regional differences in the expression pattern of opioid receptors and that the dysregulation of opioidergic signaling (including, most likely, a prolonged, pathological activation of MOR at the periphery) may contribute to the development of itch in (at least a subset of) AD patients.

In line with the above observations (i.e., that antagonizing MOR at the periphery may be crucial in some cases in the development of the anti-pruritic effects), Sullivan and Watson reported the case of a 52-year-old female patient suffering from intense, generalized pruritus associated with mycosis fungoides stage IIb. The pruritus of this patient was relieved using subcutaneous naloxone (0.2 mg at night and repeated at 3–4 h intervals as required). Intriguingly, however, the itch intensified within 30 min, when subcutaneous naloxone was changed to orally applied naltrexone (50 mg) [241].

Besides the MOR-preferring antagonist naltrexone, the highly selective KOR agonist nalfurafine was also assessed in several studies. Indeed, nalfurafine (2.5–5 µg/day p.o.) was found to be effective in a placebo-controlled clinical trial in alleviating uremic pruritus [242], and it has already been licensed in Japan to treat this condition [243].

Importantly, long-term efficacy of oral nalfurafine (2.5–5 μg/day (starting dose: 5 μg/day) p.o., reaching a plasma level of 6.19 ± 3.43 pg/mL 16 ± 4 h after the administration) was also demonstrated in hemodialysis patients suffering from intractable pruritus within the course of a 52-week open-label trial (originally, 211 patients were recruited, but only 145 completed the study). The authors found that orally applied nalfurafine was able to suppress pruritus continuously for 52 weeks without attenuation or tolerance, and itch intensified again in the post-observation period (4 weeks after the termination of treatment). However, it is important to mention that adverse effects occurred in 98.1% of the patients (207 patients), whereas adverse drug reactions were detected in 48.8% of the patients (103 patients) during the treatment. Among the side effects, insomnia and constipation were the most frequent. However, eczema (three patients) and, quite surprisingly, pruritus (seven patients) were also reported [244].

The above data clearly demonstrated the efficiency of nalfurafine-mediated KOR agonism in alleviating itch; however, the potential side effects also raised a need for more selective, ideally topical, treatments. From this perspective, it seems to be very important to mention that certain pieces of evidence argue that the anti-pruritic potential of the opioidergic signaling stems not only from its effects on the central nervous system, but also from peripheral actions on various non-neuronal cell types of the skin.

Indeed, the expression of KOR was significantly decreased in the epidermis of hemodialysis patients suffering from uremic pruritus, whereas MOR immunoreactivity did not change [245]. Moreover, KOR, but not MOR, was found to be downregulated in the lesional epidermis of patients suffering from psoriasis, and the downregulation was even more pronounced in case of itch [246]. These findings highlight the possibility that homeostatic KOR signaling on epidermal keratinocytes may negatively regulate the release of itch mediators from these cells. Moreover, it was also hypothesized that, due to enhanced receptor internalization, MORs of the epidermal keratinocytes are not accessible for the endogenous ligands (most importantly for β-endorphin) in chronically itchy skin. Thus, MOR expressed on the pruriceptive afferent nerve endings has no competition for the ligand, leading to enhanced itch sensation [193,240,247].

Last, but not least, it should also be noted that orally applied difelikefalin (a highly KOR-selective synthetic peptide [248]; 0.25–1 mg twice daily) was able to significantly alleviate the pruritus of AD patients, and it was found to be well tolerated in a multicenter, double-blind, placebo-controlled, randomized, phase 2 clinical trial (NCT04018027) [249].

#### 2.5.4. Cooperation between the Opioidergic Signaling and Certain “Itch Pathways”

It has already been shown that endothelin-1 (ET-1; a tissue-injury-associated pain and itch mediator) is one of the potential upstream regulators of β-endorphin release in keratinocytes. Indeed, in male Sprague Dawley rats, ET-1 was found to trigger the release of β-endorphin from epidermal keratinocytes. The effect was mediated via the activation of ET_B_ receptors expressed on the said cells. Indeed, IRL-1620 (ET_B_ agonist; 200–400 nM; 1 h) significantly increased β-endorphin release from primary human epidermal keratinocytes (secretion rates: 6.8 ± 0.3 pg/10^5^ cells/h (control) vs. 34.0 ± 7.1 pg/10^5^ cells/h (200 nM) and 61.7 ± 27.6 pg/10^5^ cells/h (400 nM)) in an ET_B_-dependent manner, since the ET_B_-specific antagonist BQ-788 (10 μM) prevented the action [250]. In turn, β-endorphin activated MOR and KOR on nociceptive neurons and induced hyperpolarization, most likely through the activation of G-protein-coupled inwardly rectifying potassium (GIRK, also known as Kir-3) channels. This inhibited ET-1-induced pain, which was the consequence of the activation of ET_A_ receptors on the nociceptors (unfortunately, the scratching behavior was not investigated) [250]. In light of the above data, ET-1 may act as an autocrine/paracrine regulator of β-endorphin, since keratinocytes were shown to be able to release it upon certain stimuli. Indeed, the TLR3 activator poly-(I:C) (1 μg/mL; 24 h) has recently been found to upregulate TLR3 on (Q-PCR) and to induce the release of ET-1 (ELISA) from primary human epidermal keratinocytes in a TLR3-dependent manner, since the latter effect could be abrogated by the co-administration of the TLR3 antagonist CU CPT 4a (30 μM) [251]. Moreover, TLR3 was found to be greatly upregulated in the lesional epidermis of certain pruritic dermatoses, including AD (as well as psoriasis and prurigo nodularis). This suggests that overactivation of the TLR3–ET-1 axis may play a role in the itch–scratch cycle, and hence, in the pathogenesis of these diseases [251], as well as in the elevation of blood β-endorphin levels of AD patients (see below).

Importantly, the TLR3–ET-1 axis is not the sole positive upstream regulator of β-endorphin secretion of epidermal keratinocytes. Indeed, IL-31 (300–1000 ng/mL; 24 h), a highly “AD-relevant” cytokine mostly produced by several immune cells [252], increased β-endorphin release from primary human epidermal keratinocytes via activating signal transducer and activator of transcription (STAT)-3, and a significant correlation in the blood levels of IL-31 and β-endorphin was also demonstrated in AD patients [142]. Moreover, IL-31 (1–100 ng/mL; 24 h treatments) was found to increase the production and release of T_h_2 cytokines (IL-4, IL-5, and IL-13) in PBMCs of children suffering from allergic rhinitis [253]. Furthermore, IL-31 receptor A (IL-31RA) and β-endorphin co-localized and showed higher expression in the epidermis of AD patients belonging to both extrinsic and intrinsic endotypes as compared to healthy controls [254]. Importantly, IL-31RA was recently shown to be involved in the development of morphine-induced (5 mg/kg; s.c.; C57BL/6 mice) long-lasting scratching, as this phenomenon did not develop in IL-31RA^−/−^ mice [255]. Finally, in line with the above findings, transcription of the β-endorphin precursor POMC gene was found to be positively regulated by IL-31 (50 ng/mL; 6–24 h) via the activation of ERK1/2, JNK, and p38 MAPK, and early growth response 1 (EGR-1) in HaCaT keratinocytes [254].

Importantly, opioids are not merely downstream mediators of the IL-31 signaling. As mentioned above, in a DNFB-induced contact dermatitis model of male Swiss Webster mice, the KOR agonist and MOR and DOR partial agonist (i.e., technically a MOR- and DOR-blocker) Nalbuphine (10 mg/kg; s.c.; 20 min prior to DNFB) was found to attenuate pruritus. Moreover, Nalbuphine also decreased IL-31 (from ~6 to ~4 ng/mL; ELISA) and increased IL-10 (Q-PCR) levels in the cervical skin of the animals [152]. Likewise, IL-31 expression as well as scratching were found to be suppressed by WOL071-007 (a selective KOR agonist) in an imiquimod-induced psoriasis-like cutaneous inflammation in BALB/c mice [156].

#### 2.5.5. Contradictory Data Regarding the Role of Opioidergic Signaling in Itch

Importantly, however, certain pieces of evidence suggest that the role of opioidergic signaling in the pathophysiology of itch may be even more complex and context dependent. In a dry-skin dermatitis model (evoked by twice-a-day 15 s treatment with a mixture of acetone and diethylether (1:1) for 5 days), MOR^−/−^ animals tended to scratch less (determined by scratch bouts per minute) as compared to the wild-type mice, whereas the difference was significant in the case of KOR^−/−^ animals [120]. This means that, at least in certain types of pruritus, not necessarily KOR agonism, but rather the suppression of KOR activity may be beneficial, while a blockade of MOR seems to be efficient in these cases as well. Further studies are therefore encouraged to unveil the molecular details of the anti-pruritic and putative pro-pruritic effects of KOR.

### 2.6. Opioidergic Signaling and Stress

According to common wisdom, the skin is “the mirror of the soul” [256]. Indeed, countless clinical observations demonstrate that psychoemotional stress can contribute to the appearance and/or worsening of certain “psychocutaneous” [257] skin conditions, including acne or AD [257,258,259,260,261]. Moreover, studies aiming at unveiling the underlying mechanism highlighted that the hypothalamic—pituitary—adrenal cortex (HPA) stress hormone axis is likely to play a key role in this process, while endogenous opioidergic signaling (most especially β-endorphin) was shown to regulate and fine tune the stress response [143].

Importantly, the skin is not merely the target of the HPA axis, but it can also serve as the source of these hormones [262], among which corticotropin-releasing hormone (CRH) appears to be an important link between stress and the worsening of AD [258]. This seems to be particularly important, since β-endorphin and Met-enkephalin (OGF) can be cleaved from POMC; thus, CRH production (systemic as well as local) is one of the key regulators of cutaneous opioidergic signaling [262]. Importantly, serum CRH level was found to be elevated in untreated AD (31.0 ± 19.5 pg/mL) as well as in psoriasis (22.5 ± 13.7 pg/mL) as compared to healthy individuals (9.7 ± 4.2 pg/mL), while CRH-1 receptor was downregulated in the lesional epidermis of the said patients (immunohistochemistry) [263].

Moreover, several studies assessed the effects of acute stress. Indeed, stress induced by strong exercise (25 m/min for 90 min) exacerbated dermatitis in 7-week-old male NC/Nga mice, while mild exercise (20 m/min for 60 min) reduced it. The plasma levels of α-MSH, TGF-β, and substance P were increased in parallel with the symptoms and were further elevated by strong exercise, whereas mild exercise decreased them. Mild exercise, however, increased plasma β-endorphin level, whereas serum ACTH, IgE, and TNF-α levels were not influenced [264].

Later, using the same approach (i.e., “mild” or “strong” exercise on 7-week-old male NC/Nga mice kept either under specific pathogen-free environment (SPF mice; no AD-like dermatitis) or conventional conditions, in which case AD-like dermatitis develops), the same group continued exploring the effects of stress in AD. They showed that plasma α-MSH level as well as the number of melanocortin receptor 1 positive dermal cells were increased by strong exercise but were reduced by mild exercise in conventional mice (i.e., not kept in SPF environment) [265]. On the other hand, plasma β-endorphin level was elevated in parallel with the development of the symptoms in mice kept under conventional conditions, and this elevation was further enhanced by mild exercise but suppressed by strong exercise. Moreover, mild exercise also increased the number of MOR-positive cells in the dermis [265]. Finally, the expressions of prohormone convertase (PC) 1/3 and -2 (these enzymes are sequentially involved in the production of β-endorphin [84]) as well as of carboxypeptidase E (CPE; an enzyme required for the activation of β-endorphin [85,86]) were higher in the pituitary gland of the conventional group as compared to the mice kept under SPF conditions. Intriguingly, the expression of PC2 in the conventional group was suppressed by mild, and enhanced by strong exercise [265], whereas the expression CPE was increased by mild, and suppressed by strong exercise [265].

Taken together, although there are several open questions, these data suggest that, besides the aforementioned ET-1- and IL-31-coupled signaling pathways, CRH overproduction may also contribute to the elevation of β-endorphin levels in AD, providing a possible biological link between stress and the exacerbation of AD.

### 2.7. Outside the Box: Relationship with Cannabinoid Signaling and Electroacupuncture

Intriguingly, certain data argue that opioids may serve as downstream effectors for cannabinoid signaling [5]. Indeed, intraplantar administration of the CB_2_ agonist AM1241 (10 µM; 1 h) increased β-endorphin release from keratinocytes (ca. 500 pg/mL vs. 1100 pg/mL) [266], and the effect could be prevented by the CB_2_ antagonist AM630 (1 μM). Next, the released β-endorphin activated neuronal MOR and inhibited nociception in rats [267]. Moreover, in another study, the greatly CB_2_-selective, plant-derived “cannabinoid-like” compound β-caryophyllene [268] (18 µg; i.pl.) also attenuated capsaicin-induced nociceptive response, most likely by stimulating keratinocyte-derived β-endorphin release [269]. Thus, CB_2_ seems to be a potent positive upstream regulator of β-endorphin production of epidermal keratinocytes.

Another interesting observation was made in a Sprague Dawley rat model of capsaicin (50 mg/kg s.c. 48 h after birth)-induced dermatitis. Here, high-frequency (120 Hz; 9.5 mA) electroacupuncture (30 min intermittent (10 s on; 5 s off)) treatment at specific locations every other day for 3 weeks alleviated dermatitis score of AD-like skin lesions and enhanced the production of dynorphin A(1–13) in the spinal cord (determined by Western blotting of the tissue). Intriguingly, no effect on the scratching behavior was found [270].

### 2.8. Direct Evidence: Findings in AD

Besides the above indirect clues, several lines of direct evidence also indicate that dysregulation of cutaneous opioidergic signaling may contribute to the pathogenesis of AD, and targeting this signaling system may lead to beneficial therapeutic effects. Indeed, β-casomorphin-7 (BCM7, Tyr-Pro-Phe-Pro-Gly-Pro-Ile; a bioactive cow-milk peptide derivative that plays a role in the development of cow-milk protein allergy) was shown to elevate MOR mRNA expression in PBMCs (5-day treatment of the isolated cells; 1–1000 ng/mL) of children with AD (62 children) but not in PBMCs of healthy children (40 subjects). Thus, it appears that AD patients may be predisposed to having a dysregulation in their opioidergic signaling that can be triggered by e.g., nutritional factors [33]. Importantly, because the activation of MOR on PBMCs induces a T_h_1 to T_h_2 shift [170], these findings provide a putative link between consumption of certain nutraceutical factors (e.g., cow milk) and the development of atopic phenotype.

Moreover, the serum level of β-endorphin was found to be elevated in children suffering from AD with pruritus (21 subjects; 14.95 ± 2.75 pM) compared to the healthy controls (25 subjects; 8.85 ± 2.39 pM). Interestingly, β-endorphin level of symptom-free AD patients (i.e., those who have formerly been diagnosed with AD but showed no symptoms at the time of the study) with no pruritus (20 subjects; 9.4 ± 2.46 pM) was not significantly different from the one of healthy controls [271]. In line with these observations, another group reported elevated concentration of β-endorphin in the serum of patients with severe AD (9.2 ± 3.4 ng/L, i.e., ~2.66 ± 0.98 pM vs. 6.1 ± 1.5 ng/L, i.e., ~1.76 ± 0.43 pM) [139]. Moreover, they also found a correlation between the elevated β-endorphin concentration and some clinical parameters of the disease; patients with widespread AD lesions involving more than 20% of the skin surface as well as having a high disease severity score or a previous history of bronchial asthma had higher β-endorphin levels [139]. Likewise, according to a more recent study, serum β-endorphin concentration was not only elevated in AD patients (ca. 12 pg/mL, i.e., ~3.46 pM vs. ca. 6 pg/mL, i.e., ~1.73 pM), but, alongside with trans-epidermal water loss and serum IgE level, it was suggested being used as independent biological marker for the disease severity and itch in AD [140].

As mentioned above, maybe partly because of the itch–scratch cycle, the TLR3-ET-1 axis seems to be overactivated in the lesional skin of AD patients (as well as in some other pruritic dermatoses) [251]. Moreover, ET-1, together with the highly AD-relevant cytokine IL-31, and the stress response master regulator CRH, were shown to greatly enhance β-endorphin release of epidermal keratinocytes [142,250,254,263]. Thus, one might speculate that the elevated serum β-endorphin level often detected in AD patients might be the consequence and the final common effector of the pathological activity of IL-31, CRH, and/or the TLR3–ET-1 axes, at least in a subset of AD patients.

However, it should also be noted that a prior study did not find significant differences in the β-endorphin levels of healthy individuals and AD patients. In the said study, cortisol, ACTH, and β-endorphin responses to CRH were measured in patients suffering from moderate to severe AD (additional inclusion criteria: no systemic corticosteroids in the last 3 months and no topical corticosteroid treatment in the last week), as well as in age- and sex-matched healthy individuals [141]. The circadian rhythm of cortisol secretion did not differ between the groups. However, the net response to CRH (100 µg; i.v. bolus) was significantly attenuated for both cortisol and for ACTH in the patient group, whereas the β-endorphin response did not differ between the groups (pre-stimulation level: ~4 pM; peak around 30 min: ~8 pM; in both cases) [141]. One can therefore conclude that the elevation of the β-endorphin may be absent in some AD patients. Moreover, in light of the aforementioned animal data on the effect of mild- and strong-exercise-induced stress [264,265], one might speculate that the elevation of serum β-endorphin (if present) may be an attempt of the body to cope with stress and to restore homeostasis.

Importantly, contradictory data are available about the most obvious (yet not the sole; Figure 2) receptor of β-endorphin, i.e., MOR, in AD. Indeed, MOR was found to be downregulated in the lesional epidermis of patients suffering from chronic AD, and the remaining receptors were mostly localized intracellularly, i.e., they were most likely unavailable to their peptide ligands [247]. Moreover, an analysis of the layer dependence of the MOR mRNA expression pattern revealed a shift in the expression from the suprabasal (healthy skin) toward the subcorneal (lesional skin of AD patients) layers [247], which might be caused by increased keratinocyte turnover. Intriguingly, immunoreactivity of KOR, MOR, and DOR was elevated in keratinocytes as well as in fibroblasts in hypertrophic scar tissue in situ [127].

On the other hand, another study yielded somewhat different results. Indeed, MOR-1A (COOH-terminal amino acid sequence: LENLEAETAPLP), but not MOR-1B (COOH-terminal amino acid sequence: TVDRTNHQKIDLF), was detected on the sensory nerve fibers as well as in all layers of the epidermis of human skin [272]. Intriguingly, the staining pattern did not change in AD patients, except that MOR-1A positivity of the intraepidermal nerve fibers was only occasionally observed in specimens of AD patients, as revealed by the analysis of the skin specimens of 10 healthy individuals and 5 AD patients [272]. Likewise, another study also failed to show any alteration in the epidermal expression of MOR between healthy individuals and AD patients [103]. However, it should also be noted that in these studies, MOR was found to be expressed in the whole epidermis [103,272] and not only in the suprabasal layers, where it was seen in previous studies.

Importantly, as mentioned above, the highly “AD-relevant” cytokine IL-13, as well as other factors (e.g., cow-milk-derived BCM7) were already shown to upregulate MOR expression in certain cell types [33,147]. Thus, one might speculate that, at least in a subset of AD patients, IL-13 and/or BCM7 may prevent MOR from undergoing the usual activation-induced downregulation (most likely evoked by the usually elevated β-endorphin level; see above). This may lead to a pathologically prolonged MOR activity, perhaps not only in the epidermis, but also on other cell types, including PBMCs. This may skew the immune responses toward a T_h_2-dominant direction [170], which is a commonly observed phenomenon in AD [1,2].

Finally, as mentioned above, the MOR-preferring antagonist naltrexone (1% formulation applied topically) was highly efficient in reducing itch in AD patients according to a double-blind, placebo-controlled trial involving 40 patients [240]. Importantly, the authors reported that MOR was downregulated in the lesional skin of AD patients; however, they did not investigate sex-, age- and region-matched skin specimens from healthy subjects simultaneously in the same staining procedure, leaving the degree of actual decrease questionable. Importantly, however, they showed that MOR expression was increased compared to the initial level upon a 2-week-long topical naltrexone treatment (the second sample was taken from the same region, ca. 1 cm away from the first one) [240].

In contrast to MOR, KOR expression was found to be significantly lower in AD patients, and PUVA therapy (0.6 mg/kg 8-methoxypsoralen p.o., 2–6 J/cm^2^ UVA) did not influence its staining intensity [103]. Moreover, the authors also demonstrated that, irrespective of the said PUVA treatment, epidermal immunoreactivity of β-endorphin was not significantly different between healthy individuals and AD patients [103]. On the contrary, the expressions of the rather “KOR-preferring” dynorphin A (1-8) and dynorphin A (1-17) were decreased in the epidermis of AD patients, and their levels were partially normalized by the PUVA therapy [103]. Although these data indicated that KOR was downregulated in AD lesions, other authors found that epidermal KOR expression was higher in the lesional skin of AD patients (n = 37) as compared to age- and sex-matched healthy controls (n = 24) [273] (note that these latter data have only been published in the form of a citable abstract). Collectively, these findings raised the exciting possibility that the loss of homeostatic intraepidermal KOR signaling may contribute to the onset of the symptoms of AD. In line with this hypothesis, preliminary data (reported only in the form of citable abstracts) indicate that the topically applied KOR agonist WOL071-007 ameliorated inflammation and reduced scratching frequency in a mouse model of AD in a KOR-dependent manner, since the beneficial effects did not develop in KOR^−/−^ animals [274]. Importantly, the same group also demonstrated that topically applied WOL071-007 ameliorated itch and cutaneous inflammation in AD patients in a small, placebo-controlled phase Ib study (NCT02576093) [274,275]. Moreover, as mentioned above, the selective [248] synthetic KOR-agonist difelikefalin (0.25–1 mg twice daily; p.o.) significantly suppressed pruritus of AD patients, and it was well tolerated in a multicenter, double-blind, placebo-controlled, randomized, phase 2 clinical trial (NCT04018027) [249].

Finally, it is important to mention that a variable nucleotide repeat (VNTR) polymorphism, composed of 1–4 repeats of a 68-bp element, which contains one binding site per repeat for the transcription factor activator protein (AP)-1, was identified in the promoter region of the PDYN gene (i.e., the precursor of several “KOR-preferring” ligands, including dynorphin A (1-8) and dynorphin A (1-17) (Figure 2)) [276]. Alleles 1 and 2 (one or two repeats) were classified together as “L alleles” (low expression: activation of the AP-1 complex leads to no increase over basal conditions), whereas alleles 3 and 4 (three or four repeats) as “H alleles” (high expression: pronounced increase in gene expression upon activation of the AP1 complex) [276]. Importantly, genotyping of 211 Austrian patients with AD and 197 nonatopic controls revealed that 8.5% (AD) and 9% (controls) of them were homozygous for the L alleles, 45.5% (AD) and 48% (controls) homozygous for the H alleles, and 46% (AD) and 43% (controls) were heterozygotes for the H alleles. Thus, there was no detectable association between any of the PDYN variants and AD in general. The analysis of possible associations with pruritus intensity also showed no relevant difference in the allelic distribution between patients with different pruritus score values [277]. Finally, no association between PDYN 946C → G SNP and pruritus was identified in an extended cohort of 267 Austrian AD patients [277].

Besides KOR, dysregulation of DOR signaling may also play a role in AD. Indeed, desmoglein (DSG)-1 was found to be downregulated in the lesional epidermis of AD patients, and its expression could also be suppressed experimentally in primary human epidermal keratinocytes by a highly “AD-relevant” [145,146] cytokine cocktail (IL-4 and IL-13; both at 50 ng/mL for 48 h) [278]. Importantly, the expressions of DSG-1 and -4 were shown to be elevated in the epidermis of DOR^−/−^ mice (immunofluorescent labeling), and DSG-1 was found to be downregulated in DOR overexpressor human N/TERT-1 keratinocytes (Q-PCR) [119]. Moreover, 12 h Met-enkephalin (OGF; 10 nM) treatment of DOR overexpressor human N/TERT-1 keratinocytes altered the intracellular localization of desmoplakin, leading to a punctate distribution at the periphery of the cells where cell–cell contacts were weakened [119]. Thus, these data highlight the possibility that abnormally increased DOR activity may contribute to the damage of the physicochemical barrier in AD, especially since besides MOR, β-endorphin can activate DOR as well (Figure 2).

## 3. Discussion: Challenges, Open Questions, Promising Research Directions

### 3.1. Promising Possibilities: How Could Modulation of the Opioidergic Signaling Alleviate AD?

**Figure 3:**
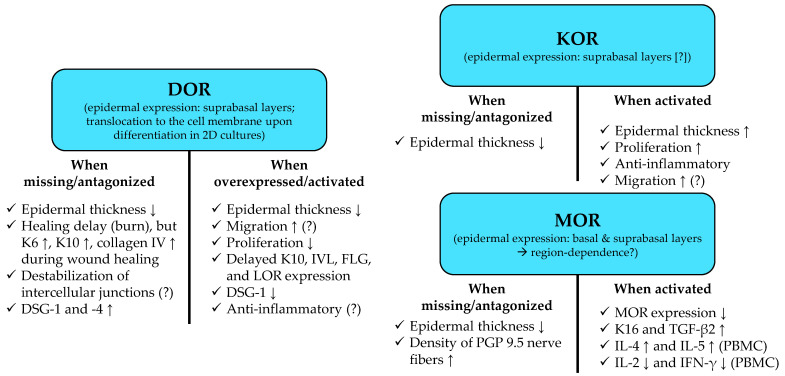
Overview of the expression patterns and “AD-relevant” roles of the major opioid receptors in human epidermal keratinocytes and PBMCs. DOR, KOR, and MOR are involved in the regulation of the proliferation/differentiation balance of the epidermal keratinocytes, as well as in influencing immune responses. (Question marks indicate conflicting data/unclear effects.)

As discussed above in detail, opioidergic signaling is deeply involved in regulating several AD-relevant aspects of cutaneous (patho)physiology (Figure 3). Indeed, the major opioid receptors were all shown to be functionally expressed at various compartments of the human skin and to regulate e.g., proliferation and differentiation balance of the epidermal keratinocytes, local immune responses, or itch. Moreover, the activation of MOR on PBMCs promotes a shift toward a T_h_2-type immune response that can be characterized by the increased production of several “AD-relevant” cytokines, including e.g., IL-4, IL-5.

**Figure 4:**
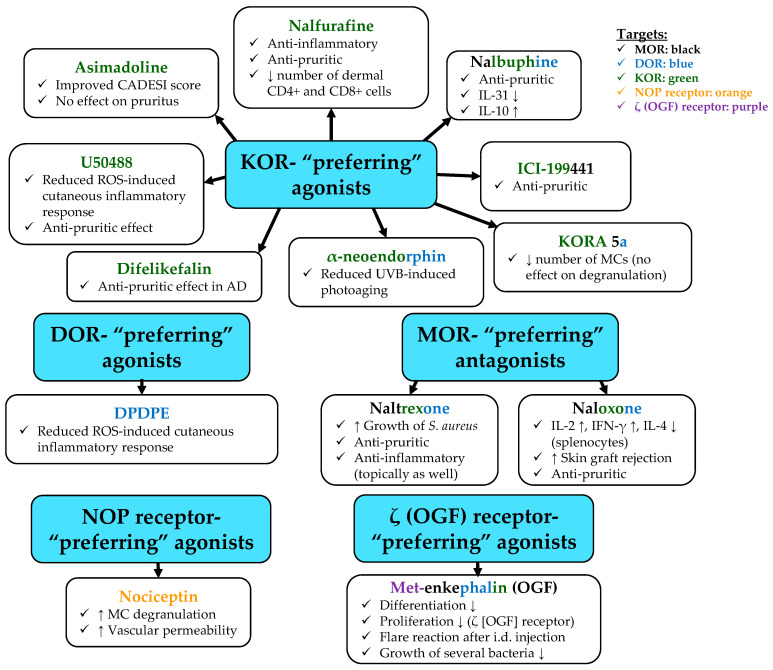
Overview of the “AD-relevant” effects of selected modulators of the key opioid receptors. Note that most of the above compounds are not selective (for details, see Figure 2) but can rather concentration dependently activate/antagonize multiple opioid receptors. The color code indicates the relevant targets of each molecule. When one molecule can activate or antagonize more than one receptor, the order of the colors from the left to the right represents the order of the affinity toward each receptor. For example, the order of colors in the case of “**Naltrexone**” (**black-green-blue**) indicates that its affinity is highest with human MOR, it is less with KOR, and even less with DOR. Affinity data are obtained from the IUPHAR Guide to Pharmacology database (accessed on 5 December 2021) as well as from Ref [121] (in the case of KORA 5a). Importantly, lack of a specific color does not necessarily mean lack of the respective binding but rather *lack of experimental evidence* of the said binding. Please note that **we only provide affinity but not efficacy data** of the substances. This is especially important in the case of Nalbuphine, since it can simultaneously activate KOR, and, as a partial agonist, suppress full agonist-induced MOR and DOR signaling.

This obviously means that differential modulation of each receptor would be most desirable in managing AD. Indeed, the activation of KOR as well as pharmacological blockade of MOR promise to exert potent anti-pruritic as well as anti-inflammatory effects. Moreover, these measures may also counterbalance the T_h_2-skewed immune phenotype of AD patients (Figure 4). Theoretically, DOR activators as well as NOP receptor blockers may also exert beneficial anti-inflammatory effects e.g., by preventing MC degranulation (Figure 4). Moreover, appropriate modulation of ζ (OGF) receptor, as well as administration of Met-enkephalin (OGF) are also likely to improve epidermal differentiation, and hence, the physicochemical barrier function. Finally, Met-enkephalin (OGF) may influence the composition of the cutaneous microbiota as well (Figure 4).

### 3.2. β-Endorphin: A Key Molecule That Connects Several AD-Relevant Signaling Pathways

**Figure 5:**
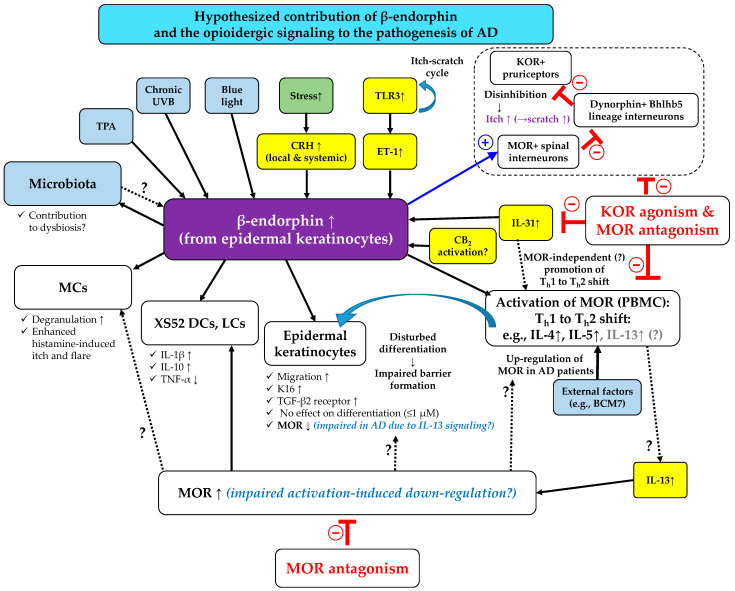
Hypothesized contribution of β-endorphin and IL-13-induced upregulation (or impaired activation-induced downregulation) of MOR to the pathogenesis of AD. Blue background marks external factors, internal contributors are highlighted with yellow background, whereas green background indicates that stress can be the consequence of internal and external causes as well. As shown on the figure, β-endorphin appears to be a common point in multiple AD-relevant pathways. Although there is a continuous β-endorphin-mediated activation, at least in a subset of the AD patients, MOR does not downregulate, most likely because of the presence of IL-13 and/or certain external factors (e.g., BCM7). This may lead to pathologically prolonged MOR activity further skewing the immune response toward T_h_2 polarization, and possibly disturbing differentiation of epidermal keratinocytes, as well as maturation/degranulation of cutaneous MCs. However, it should also be noted that certain data argue for a different working hypothesis. Indeed, as mentioned in the main text, certain reports argue that expression pattern of MOR alters in certain AD patients, i.e., MOR becomes intracellular in the keratinocytes of the lesional skin, which leaves MOR on the sensory neurons without competition for the ligand (β-endorphin), leading to overactivation of the said neuronal receptors [193,240,247]. (Question marks indicate hypothesized effects.)

As mentioned above, several (yet, not all) studies found elevated β-endorphin levels in the serum of AD patients [139,140,271]. Moreover, epidermal keratinocytes are not only able to release biologically relevant amounts of this endogenous opioid, but they can do it in response to a wide variety of “AD-relevant” and other stimuli, including CRH, ET-1, IL-31, CB_2_ activation, UVB and blue light irradiation, or TPA treatment (Figure 5).

As mentioned above, the subsequently elevated β-endorphin level may be part of the human body’s strategy to cope with various stressors and to restore homeostasis [264,265]. However, it can also lead to several AD-relevant (mostly MOR-related) biological events, including e.g., MC degranulation, T_h_1 to T_h_2 shift in the immune response, itch, or (theoretically) even cutaneous dysbiosis (Figure 5).

Importantly, despite the continuously elevated β-endorphin levels in the serum, no MOR downregulation can be observed in most of the AD patients. Because the highly “AD-relevant” cytokine IL-13 as well as certain nutraceutical factors (e.g., cow-milk-derived BCM7) were shown to upregulate MOR in certain cell types, one might speculate that, at least in a subset of the patients, MOR expression may remain high, and the subsequently sustained pathological β-endorphin → MOR signaling may contribute to the pathogenesis of AD at multiple levels (Figure 5).

Indeed, the activation of MOR on PBMCs was shown to skew the immune response toward a T_h_2-dominant direction. Characteristic T_h_2 cytokines (e.g., IL-4) as well as the MOR-induced itch–scratch cycle can impair the epidermal physicochemical barrier, and the said pathologically prolonged MOR activity may also contribute to the damage of the barrier. Because KOR agonism and MOR antagonism both appear to exert potent anti-pruritic as well as anti-inflammatory effects, one might speculate that therapeutic agents combining these approaches (e.g., Nalbuphine) may be especially potent in improving AD (Figure 5).

### 3.3. Unexplored Territories, Contradictions, Upcoming Challenges

As discussed above, the exploration of (cutaneous) opioidergic signaling is a challenging task due to several reasons. The most important issues that are to be solved in the future include, but are not limited to:

- Lack of KO-validated, subtype-specific antibodies
- Insufficient knowledge on the expression pattern and putative differential role, as well as ligand selectivity of the splice variants of the major receptors
- Signaling bias as well as receptor heteromerization
- Lack of receptor-subtype-selective agonists and antagonists
- Lack of sufficient knowledge on the biological characteristics of the splice variants of the endogenous ligands
- Unexplored subcellular distribution (surface vs. unavailable intracellular receptor pools)
- Regional and sex differences in the expression pattern of the receptors and ligands

Finally, it is also important to emphasize that this review could not cover all aspects of the pathogenesis of AD. Thus, the interactions between opioidergic signaling and these “other aspects”, including, but not limited to, the role of the mediators released from epidermal sensory nerve endings, the microvasculature, members of the innate immune system, etc., also deserve to be explored in future, targeted studies.

## 4. Concluding Remarks

Although, as mentioned above, several open questions await to be answered, the available evidence clearly demonstrates that the dysregulation of opioidergic signaling may be involved in the pathogenesis of AD (in at least a subset of the patients). Moreover, KOR agonism as well as MOR antagonism appear to be potent tools in alleviating AD-related itch and in improving cutaneous symptoms. Further studies are therefore encouraged to unveil whether a combination of these approaches can efficiently complement the already existing therapeutic possibilities in AD.

## Data Availability

Not applicable.

## Abbreviations

5′GNTI	5′-guanidinonaltrindole
ACTH	adrenocorticotropic hormone
AD	atopic dermatitis
AITC	allyl isothiocyanate
AP-1	activator protein-1
BCM7	β-casomorphin-7
CADESI	Canine Atopic Dermatitis Extent and Severity Index
cAMP	cyclic adenosine monophosphate
CB_2_	cannabinoid receptor 2
CCK	cholecystokinin
CCL2	C-C motif chemokine ligand 2, also known as monocyte chemoattractant protein 1
CPE	carboxypeptidase E
CREB	cAMP response element-binding protein
CRH	corticotropin-releasing hormone
CXCL1	chemokine (C-X-C motif) ligand 1, also known as keratinocyte-derived cytokine
DMSO	dimethyl sulfoxide
DNFB	dinitrofluorobenzene
DOR	δ-opioid receptor
DRG	dorsal root ganglia
DSG	desmoglein
EGR-1	early growth response 1
ERK	extracellular signal-regulated protein kinase
ET	endothelin
EV	extracellular vesicle
FLG	filaggrin
G-CSF	granulocyte colony-stimulating factor
GIRK	G-protein-coupled inwardly rectifying potassium, also known as Kir-3
GRP	gastrin releasing peptide
GRPR	gastrin releasing peptide receptor
HPA	hypothalamic—pituitary—adrenal cortex
i.d.	intradermal
IFN	interferon
IL	interleukin
IL-31RA	interleukin-31 receptor A
JNK	c-Jun N-terminal kinase
K	keratin
KC	keratinocyte-derived cytokine, also known as chemokine (C-X-C motif) ligand 1
KLH	keyhole limpet hemocyanin
KOR	κ-opioid receptor
LC	Langerhans cell
LPS	lipopolysaccharide
MAPK	mitogen-activated protein kinase
MC	mast cell
MCP-1	monocyte chemoattractant protein 1, also known as C-C motif chemokine ligand 2
Mrgpr	Mas-related G-protein-coupled receptor
MIC	minimal inhibitory concentration
MIF	melanocyte inhibiting factor
MMP	matrix metalloproteinase
MOR	μ-opioid receptor
MRGPRX2	MAS-related GPR family member X2
MSH	melanocyte-stimulating hormone
mTOR	mammalian target of rapamycin
NK cell	natural killer cell
NOP receptor	nociceptin/orphanin FQ receptor
nor-BNI	nor-binaltorphimine
NPFF	neuropeptide FF
NPPB	natriuretic polypeptide b
OGF	opioid growth factor (Met-enkephalin)
p.o.	per os
p16^INK4a^	cyclin-dependent kinase inhibitor 2A
p21^WAF1/CIP1^	cyclin-dependent kinase inhibitor 1
PAR	protease-activate receptor
PBMC	peripheral blood monomorphonuclear cell
PC	prohormone convertase
PDYN	preprodynorphin
PENK	proenkephalin-A
PKA	protein kinase A
POMC	proopiomelanocortin
pP90^RSK^	90-kDa Ribosomal S6 Kinase
PUVA	psoralen-ultraviolet A
R7bp	regulator of G protein signaling 7 binding protein
RTX	resiniferatoxin
s.c.	subcutaneous
SPF	specific pathogen-free
STAT	signal transducer and activator of transcription
TGase	transglutaminase
TGF	transforming growth factor
T_h_	helper T cell
TLR	toll-like receptor
TNF-α	tumor necrosis factor-α
TPA	12-O-tetradecanoylphorbol-13-acetate
TRPA	transient receptor potential ankyrin
TRPM	transient receptor potential melastatin
TRPV	transient receptor potential vanilloid
VNTR	variable nucleotide repeat
